# Quinolinic acid impairs mitophagy promoting microglia senescence and poor healthspan in *C. elegans*: a mechanism of impaired aging process

**DOI:** 10.1186/s13062-023-00445-y

**Published:** 2023-12-20

**Authors:** Anjila Dongol, Xi Chen, Peng Zheng, Zehra Boz Seyhan, Xu-Feng Huang

**Affiliations:** School of Medical, Indigenous and Health Sciences, University of Wollongong, Northfields Avenue, Wollongong, NSW 2522 Australia

**Keywords:** Microglia, Quinolinic acid, Neuroinflammation, Mitochondria, Mitophagy, Mitolysosome, Aging, Senescence

## Abstract

Senescent microglia are a distinct microglial phenotype present in aging brain that have been implicated in the progression of aging and age-related neurodegenerative diseases. However, the specific mechanisms that trigger microglial senescence are largely unknown. Quinolinic acid (QA) is a cytotoxic metabolite produced upon abnormal activation of microglia. Brain aging and age-related neurodegenerative diseases have an elevated concentration of QA. In the present study, we investigated whether QA promotes aging and aging-related phenotypes in microglia and *C. elegans*. Here, we demonstrate for the first time that QA, secreted by abnormal microglial stimulation, induces impaired mitophagy by inhibiting mitolysosome formation and consequently promotes the accumulation of damaged mitochondria due to reduced mitochondrial turnover in microglial cells. Defective mitophagy caused by QA drives microglial senescence and poor healthspan in *C. elegans*. Moreover, oxidative stress can mediate QA-induced mitophagy impairment and senescence in microglial cells. Importantly, we found that restoration of mitophagy by mitophagy inducer, urolithin A, prevents microglial senescence and improves healthspan in *C. elegans* by promoting mitolysosome formation and rescuing mitochondrial turnover inhibited by QA. Thus, our study indicates that mitolysosome formation impaired by QA is a significant aetiology underlying aging-associated changes. QA-induced mitophagy impairment plays a critical role in neuroinflammation and age-related diseases. Further, our study suggests that mitophagy inducers such as urolithin A may offer a promising anti-aging strategy for the prevention and treatment of neuroinflammation-associated brain aging diseases.

**Supplementary Information:**

The online version contains supplementary material available at 10.1186/s13062-023-00445-y.

## Introduction

Neuroinflammation is considered to be a major contributor to the aging process, leading to a gradual decline in cellular and physiological function over time [1]. Microglia, the primary immune cells in the brain, have been identified as key players in neuroinflammation-mediated brain aging [2]. Previously, studies in the human brain have demonstrated the presence of a distinct microglial phenotype known as senescent microglia in the aged brain [3, 4]. Furthermore, the accumulation of senescent microglia has been implicated in the onset and progression of various age-related neurodegenerative diseases, including Alzheimer’s disease (AD) [4–7]. Senescent microglia are known to function abnormally, which can lead to prolonged neuroinflammation, promoting neurodegeneration and aging-related neurodegenerative diseases [8]. However, the specific mechanisms that trigger microglial senescence in normal aging and disease conditions require investigation.

Quinolinic acid (QA) is a cytotoxic metabolite produced upon abnormal activation of microglia [9, 10]. Brain aging and age-related neurodegenerative diseases, such as AD, are associated with increased concentrations of QA [11–13]. A recent study has shown the potential of QA to induce cognitive decline and poor healthspan associated with aging in various biological systems such as humans, mice, nematodes and cell cultures [14]. However, the mechanisms by which QA promotes aging and aging-related phenotypes remain unclear. QA toxicity induces mitochondrial alterations while increasing the formation of the reactive oxygen and nitrogen species [15]. As a result, QA has been previously shown to induce brain damage through mitochondrial damage [16]. Several studies revealed that QA causes alteration in mitochondrial structure and functions, such as dilatation of cristae, impaired oxygen consumption rate, increased production of reactive oxygen species (ROS), decreased mitochondrial membrane potential (MMP), and impaired oxidative phosphorylation [17–20].

Damaged mitochondria can be removed by a selective type of autophagy known as mitophagy. Mitophagy is the key mitochondrial quality control mechanism that eliminates damaged mitochondria to maintain mitochondrial homeostasis [21]. However, defective mitophagy results in the accumulation of damaged mitochondria, one of the key features of aging and age-related diseases [22–24]. In accordance, several studies have shown that mitochondrial dysfunction and mitophagy impairment are responsible for the establishment of the senescent phenotype in response to cellular stress [25, 26]. Importantly, defective mitophagy has recently been recognised as a key contributor to aging and age-related neurodegenerative diseases [23, 27]. Moreover, mitophagy restoration has been shown to extend the lifespan in *C. elegans* and delay age-associated disorders [22, 23]. Given that QA is known to be detrimental to mitochondrial structure and function, in our study, we investigated specifically whether QA impairs mitophagy and promotes age-related phenotypes in microglia and *C. elegans*.

Here, we demonstrate that QA, secreted by abnormal microglial stimulation, impairs mitophagy by inhibiting the formation of mitolysosomes in microglial cells and in the neurons of *C. elegans*. Importantly, we show that mitophagy impairment by QA leads to the accumulation of damaged mitochondria, thereby promoting age-related phenotypes such as microglial senescence and poor healthspan in *C. elegans*. Moreover, we show that oxidative stress is a potential mediator of QA-induced mitophagy impairment and the age-related phenotype in microglia. Finally, our results showed that restoration of mitophagy by urolithin A (UA) prevents the accumulation of damaged mitochondria and rescues age-related phenotypes induced by QA in both microglia and *C. elegans*. Taken together, our results reveal that the blockade of mitophagy by QA is an important aetiology underlying aging, which may play a central role in neuroinflammation-induced age-related diseases. Further, our study suggests UA as a promising anti-aging drug candidate for the prevention and treatment of neuroinflammation-induced age-related diseases.

## Materials and methods

### Cell culture and treatments

*Cell lines.* The murine BV2 microglial cell line was a kind gift from Prof. Justin Yerbury’s lab. BV2 microglial cells were maintained in DMEM/F12 supplemented with 10% FBS (Bovogen Biologicals, #SFBS), 2 mM Glutamine (Gibco, #25030149) and 1% Penicillin/Streptomycin (Gibco, #15140122) at 37 ^o^C and 95% air/5% CO_2_. HMC3 cells were purchased from ATCC (CRL-3304) and were maintained in DMEM/F12 supplemented with 10% FBS and 1% Penicillin/Streptomycin at 37 ^o^C and 95% air/5% CO_2_. To test the effects of QA, BV2 cells or HMC3 cells were treated with QA (Sigma-Aldrich, #P63204) at the indicated concentrations for indicated times. To test the effects of oxidative stress, BV2 cells were treated with 150 µM hydrogen peroxide (H_2_O_2_) (Sigma-Aldrich, #323381) for 2 h. Following the 2 h H_2_O_2_ treatment, cells were washed twice with PBS and further cultured for 72 h in the culture medium. To test the effects of amyloid β (Aβ), BV2 cells were treated with 0.6 µM Aβ_1−42_ (Cayman Chemical, #20574) for 48 h. To induce mitophagy, BV2 cells or HMC3 cells were pre-treated with UA (Cayman Chemical, #22607) at the indicated concentrations for 2 h followed by either QA or H_2_O_2_ treatment as indicated. For inhibitor treatments, RO 61-8048 (Sigma-Aldrich, #SML0233) at the indicated concentrations and 20 µM chloroquine (CQ) (Sigma-Aldrich, #C6628) were added 30 min prior Aβ_1−42_ and UA treatment, respectively.

*Primary hippocampal neurons and microglia co-cultures.* Primary hippocampal neurons were harvested from postnatal day 0–3 C57BL/6J mouse pups. At first, hippocampal regions from brain tissue were digested in papain (Sigma-Aldrich, #P4762) according to the manufacturer’s protocol. Hippocampal neurons were seeded in poly-D-lysine (Sigma-Aldrich, #P7280) coated 12-well plates in Neurobasal medium (Gibco, #21103049) supplemented with 1% B27 (Gibco, #17504044). Cultures were maintained for 14 days in vitro (DIV) at 37 ^o^C and 95% air/5% CO_2_. Culture media were changed twice weekly. For the establishment of primary hippocampal neurons and microglia co-cultures, BV2 microglial cells were harvested with 0.05% trypsin-EDTA (Gibco, #25300054) at 37 ^o^C and 95% air/5% CO_2_ for 5 min, and were resuspended in Neurobasal medium supplemented with 1% B27. BV2 cells were plated onto the primary hippocampal neurons (DIV14) at a 1∶1 microglia:neuron ratio. Aβ_1−42_ treatment and BV2 microglial cells were added simultaneously and cultured for 48 h in a Neurobasal medium supplemented with 1% B27. Following 48 h Aβ_1−42_ treatment, the conditioned medium was collected and centrifuged at 14,000 rpm for 20 min at 4 °C. After centrifugation, supernatants were collected and stored at -80 ^o^C until further use.

### Western blot analysis

Cells were lysed in NP-40 lysis buffer (Invitrogen, #FNN0021) containing a protease inhibitor cocktail (Sigma-Aldrich, #P8340), beta-glycerophosphate and phenylmethanesulfonyl fluoride (PMSF) (Sigma-Aldrich, #P7626). Protein concentration was determined using DC assay, and 10 µg of total protein was prepared in Laemmli buffer and loaded onto a 4–20% Criterion TGX Precast Gels (Bio-Rad Laboratories, #5671095) for SDS-PAGE. Following electrophoresis, the proteins were transferred to polyvinylidene difluoride membranes (Cytiva, #GE10600021). The membranes were blocked with 5% skim milk diluted in TBS (20 mM Tris, pH 7.6, 150 mM NaCl) containing 0.1% Tween20, followed by incubation with the following primary antibodies against: LC3B (Cell Signalling Technology, #2775S), PGC1-α (Santa-Cruz, #SC-13067), p62 (Cell Signalling Technology, #5114S), 3-Hydroxyanthranilic acid 3, 4-dioxygenase (3-HAAO) (Sigma-Aldrich, #SAB2101008) and β-actin (Millipore, #MAB1501) in 1% skim milk diluted in TBS (20 mM Tris, pH 7.6, 150 mM NaCl) containing 0.1% Tween 20 overnight at 4 ^o^C. Secondary antibodies were horseradish peroxidase-conjugated anti-rabbit IgG (Cell Signalling Technology, #7074S), horseradish peroxidase-conjugated anti-rabbit IgG (Invitrogen, #31460), and horseradish peroxidase-conjugated anti-mouse IgG (Millipore, #AP308P). Blots were visualised using Amersham ECL Detection Reagents (Cytiva, #GERPN2106). Chemiluminescence detection was performed using Amersham Imager 600 RGB. The bands corresponding to the proteins of interest were analysed using the automatic imaging analysis system Quantity One (Bio-Rad Laboratories). Relative protein levels in each sample were normalised to the housekeeping gene *Actb* and presented as relative protein levels to control.

### Evaluation of mitophagy in vitro using pCLBW Cox8-EGFP-mCherry reporter

Cells were transfected with pCLBW Cox8-EGFP-mCherry (a gift from David Chan; Addgene plasmid # 78520) by adding 0.5 µg of plasmid and 1.75 µl of Lipofectamine 2000 (ThermoFisher Scientific, #11668027) into each well of a 24-well plate for 24 h, and then transiently transfected cells were subjected to designated treatments. Following treatment, cells were fixed in 4% paraformaldehyde and mounted with Prolong Diamond antifade reagent (ThermoFisher Scientific, #P36961) for confocal microscopy. Images were taken using Leica SP8 confocal microscope at either 63× or 93×. The number of mCherry-only puncta (mitolysosomes) per cell was counted using the Cell Counter Plugin in Fiji.

### Immunofluorescence

For the analysis of mitochondrial mass and mitochondrial morphology, after designated treatments, cells were stained with 200 nM MitoTracker Deep Red (Molecular Probes, #M22426) for 30 min at 37 °C. Following incubation, cells were fixed in 4% paraformaldehyde and mounted with Prolong Diamond antifade reagent for confocal microscopy. Images were taken using Leica SP8 confocal microscope at 63×. The mitochondrial footprint per cell was measured using Fiji. Mitochondrial footprint refers to the total area of mitochondria stained with MitoTracker Deep Red. Mitochondrial morphology was scored into three categories as described previously [28]. Cells with complete fragmentation, resulting in only mitochondrial spheres, were placed in the “Fragmented” category. Cells with extensive fragmentation but contained some very short mitochondrial rods (< 5 μm in length) were placed in the “Intermediate” category. Cells with medium-length mitochondrial tubules (> 5 μm in length) were placed in the “fused” category, regardless of whether there was accompanying fragmentation or not.

### Measurement of MMP

MMP was evaluated by using tetramethylrhodamine ethyl ester (TMRE) dye (Cayman Chemical, #21426) according to the manufacturer’s manual. Following treatments, cells were stained with 300 nM TMRE for 30 min at 37 °C. Cells were trypsinised, collected, washed and resuspended in ice-cold PBS. Stained cells were analysed in FL-2 channels with a BD Accuri flow cytometer. At least 10,000 events were recorded per sample. Experiments were performed in duplicate.

### Measurement of cellular and mitochondrial ROS production

Cellular ROS and mitochondrial ROS generation were evaluated with DCFDA (Cayman Chemical, #85155) and MitoSOX Red (ThermoFisher Scientific, #M36008), respectively. Following treatments, cells were stained with either 10 µM DCFDA or 3 µM MitoSOX Red for 30 min at 37 °C. Cells were trypsinised, collected, washed and resuspended in ice-cold PBS. Stained cells were analysed in FL-1 and FL-2 channels for DCFDA and MitoSOX Red respectively, with a BD Accuri flow cytometer. At least 10,000 events were recorded per sample. Experiments were performed in duplicate.

### Senescence associated-β-galactosidase (SA-β-Gal) staining

SA-β-Gal analysis was performed as previously described [29]. Briefly, following treatment, cells were fixed for 5 min in fixative solution and stained with staining solution (1 mg/ml X-gal, 40 mM citric acid/sodium phosphate, pH 6, 5 mM potassium ferrocyanide, 5 mM potassium ferricyanide, 150 mM NaCl, 2 mM MgCl_2_) for 16 h at 37 ^o^C. Cells were washed twice with PBS and imaged on Lionheart FX automated microscope. The percentage of SA-β-Gal positive cells per imaging field was quantified using the Cell Counter Plugin in Fiji.

### Quantitative real-time PCR (qRT-PCR)

Total RNA was extracted using Aurum Total RNA Mini Kit (Bio-Rad Laboratories, #7326820) and reversed to cDNA by using Applied Biosystems High Capacity cDNA Reverse Transcription Kit (ThermoFisher Scientific, #4368814). qRT-PCR was performed via SensiFAST SYBR No-ROX Kit (Bioline, #BIO-98005). For qRT-PCR, following mouse primers (all from Sigma-Aldrich) were used. ***Glb1*** Fwd 5’- GGATGGACAGCCATTCCGAT-3’, Rev. 5’-CAGGGCACGTACATCTGGATA-3’; ***p16*** Fwd 5’- GCCCAACGCCCCGAACTCTTTC-3’, Rev. 5’-GCGACGTTCCCAGCGGTACACA-3’; ***p21*** Fwd 5’-CCTGGTGATGTCCGACCTG-3’, Rev. 5’-CCATGAGCGCATCGCAATC-3’; ***p53*** Fwd 5’- ACCGCCGACCTATCCTTACC-3’, Rev. 5’-TGTCCCGTCCCAGAAGGTT-3’; ***Gapdh*** Fwd 5’- TGAAGCAGGCATCTGAGGG-3’, Rev. 5’-CGAAGGTGGAAGAGTGGGAG-3’. *Gapdh* was used as a housekeeping gene. Experiments were performed in duplicate. Relative gene expression in each sample was normalised to the housekeeping gene *Gapdh* and presented as a relative gene expression level to control.

### High-performance liquid chromatography (HPLC)

QA concentration was measured as described previously [30]. For QA standard preparation, QA was dissolved in HPLC-grade water to a final concentration of 1 µM – 200 µM. HPLC analysis was performed with a SUPELCOSIL LC-18 column (250 × 4.6 mm, 5 μm; 58298, Supelco). The mobile phase was 75% 10 mM sodium dihydrogen (pH-2.0) (Supelco, #7558-80-7) containing 25% methanol (Sigma-Aldrich, #34860), and the flow rate was 1.15 ml/min with UV (220 nm) detection. The column temperature was set at 37 ^o^C, and the injection volume was 10 µl. The retention time of QA in a conditioned medium was determined from those of standards. The amount of QA was determined from the peak area.

### Drug treatment of *C. elegans* strains

Strains used in this study are N2 wild type and SJZ42 *foxEx3 [rgef-1p::tomm-20::Rosella]* which were provided by the Caenorhabditis Genetics Center (University of Minnesota, USA). *C. elegans* strains were cultured at 20 ^o^C on standard nematode growth media (NGM) agar plates seeded with *E. coli* strain OP50 (Caenorhabditis Genetics Center) as described previously [31]. Synchronous worm populations were generated via a 6 h egg lay by gravid adults to obtain tightly synchronised L4 stage worms. Worms were treated with designated drugs from L4 stage and were transferred every 2 d to fresh drug plates seeded with *E. coli* strain OP50. QA and UA were dissolved in a DMSO stock solution and added at the indicated concentration just before pouring the plates. The control worms were treated with the corresponding concentration of DMSO at 0.6% final.

### Evaluation of mitophagy in vivo using a *C. elegans* mitophagy reporter strain

A *C. elegans* neuronal mitophagy reporter strain SJZ42 *foxEx3 [rgef-1p::tomm-20::Rosella]* was used to quantify neuronal mitophagy within anterior head neurons in worms as described previously [32]. The fluorescence intensity of the GFP signal was quantified to determine the mitophagosomes, whereas the mitolysosomes were calculated as GFP/DsRed fluorescence intensity. Thus, the lower the ratio of pixel intensity, the higher the level of mitolysosomes. Images of worms at day 5 of adulthood were taken using Leica SP5 confocal microscope at 63×. All images were acquired under the same exposure conditions.

### *C. elegans* healthspan assays

For evaluations of pharyngeal pumping rates and fertility, N2 wild-type *C. elegans* were used and performed at 20 ^o^C as described previously [22].

*Pharyngeal pumping.* Synchronised L4 stage worms per condition were transferred on NGM agar plates (10 worms per plate) seeded with *E. coli* strain OP50. At designated ages, the pharyngeal pumping was measured by counting the contraction of the pharynx manually for 30 s. The experiment was done on 9–16 individual worms randomly selected.

*Fertility.* Five synchronised L4 stage worms per condition were laid on NGM agar plates (1 worm per plate) seeded with *E. coli* strain OP50 and allowed to lay eggs for 24 h. Every 24 h, each worm was transferred onto a new plate, and this step was repeated for 4 d. The number of eggs laid was assessed 48 h after the removal of mother worms from the plates.

### Statistical analysis

Data are presented as mean ± SEM. Two-tailed unpaired *t*-tests were used for comparisons between two groups. Group differences were analysed with one-way analysis of variance (ANOVA) followed by Tukey’s multiple comparisons test or two-way repeated measures ANOVA followed by Bonferroni post-tests for multiple groups. Prism 7.0 (GraphPad Software) was used for the statistical analysis. *P* values < 0.05 were considered statistically significant.

## Results

### QA impairs mitolysosome formation

**Fig. 1:**
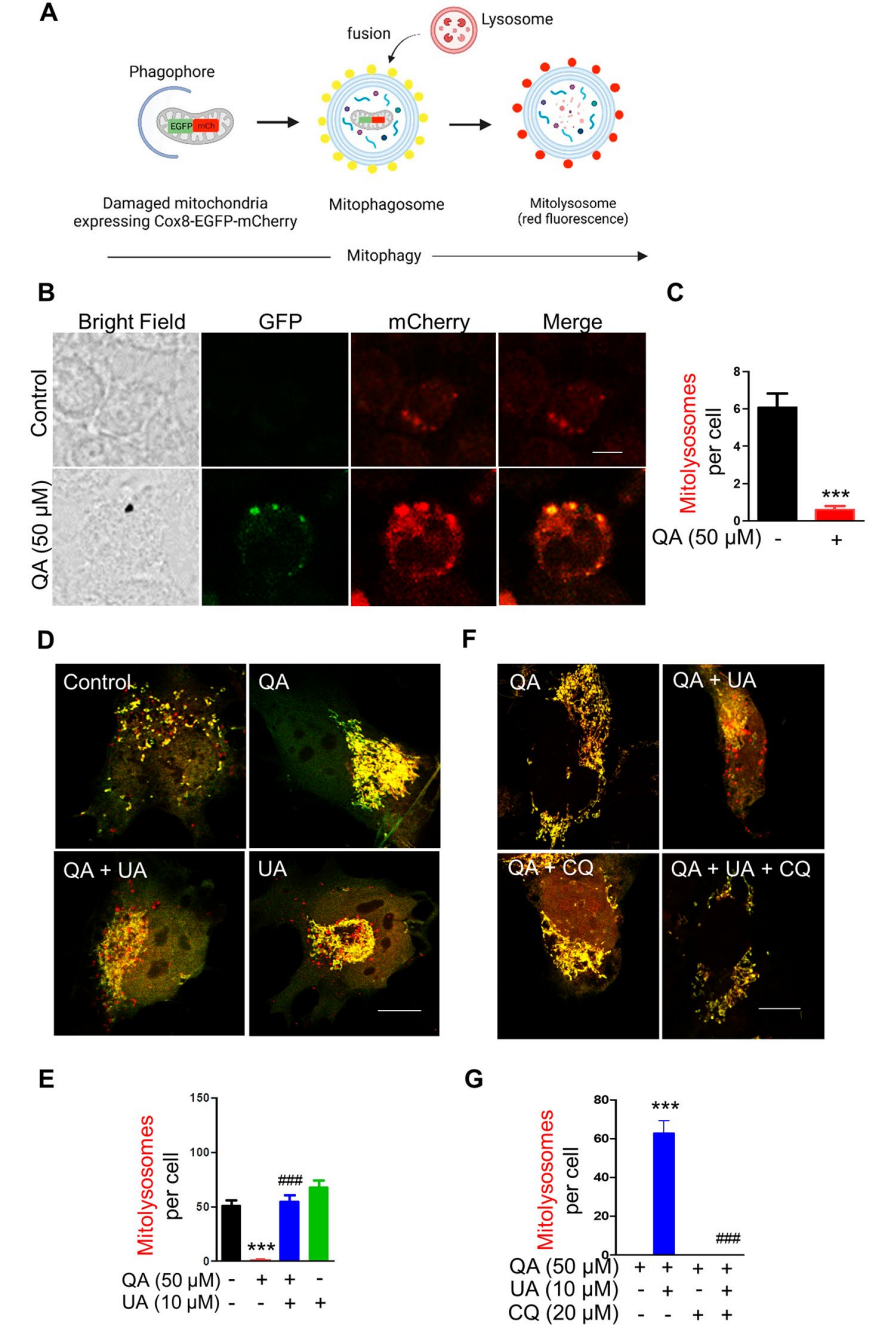
QA impairs mitolysosome formation, which is restored by mitophagy inducer UA in microglia. (**A**) Schematic working model of Cox8-EGFP-mCherry mitophagy reporter construct. Created with BioRender.com (**B**) Representative confocal images of BV2 cells transiently transfected with Cox8-EGFP-mCherry plasmid treated with or without 50 µM QA for 72 h. Scale bar, 5 μm. (**D**) Representative confocal images of HMC3 cells transiently transfected with COX8-EGFP-mCherry plasmid treated with or without 50 µM QA for 24 h in the absence or presence of 10 µM UA. Scale bar, 10 μm. (**F**) Representative confocal images of 50 µM QA-treated HMC3 cells transiently transfected with Cox8-EGFP-mCherry plasmid in the absence or presence of 10 µM UA and/or 20 µM CQ for 24 h. Scale bar, 10 μm. (**C, E**, and **G**) Graphs represent the number of mitolysosomes (red dots) per cell in indicated conditions. Data are means ± SEM. (**C**) *n* = 12–15 cells, (**E**) *n* = 10 cells, and (**G**) *n* = 8 cells; *** *p* < 0.001 versus no treatment (control) group (**C**, **E**), *** *p* < 0.001 versus QA alone treatment group (**G**), ^###^*p* < 0.001 versus QA alone treatment group (**E**), ^###^*p* < 0.001 versus QA and UA treatment group (**G**); Two-tailed unpaired *t* test (**C**) or one-way ANOVA followed by Tukey’s multiple comparisons test (**E** and **G**)

Mitophagy is a mitochondrial quality control system of cells in which double membrane structure mitophagosomes are fused with lysosomes to form mitolysosomes, where damaged mitochondrial contents are degraded (Fig. 1A) [21]. Consequently, dysfunction in mitophagy has been implicated in aging and several age-associated neurodegenerative disorders, including AD [22, 23]. Several studies reported the role of QA in the alteration of mitochondrial structure and functions [18, 19], but its regulatory role in mitophagy has been unexplored. We observed QA significantly increases the protein level of LC3B-II, an autophagosome marker, compared to the control group after 72 h treatment in BV2 cells by western blot (Additional file 1: Fig. S1A, B). However, we found that QA significantly reduced the number of mitolysosomes in BV2 cells transiently transfected with Cox8-EGFP-mCherry construct compared to the control group after 72 h of treatment (Fig. 1B, C). This indicates that the blockade of mitophagic activity by QA is due to the inhibition of fusion between mitophagosomes and lysosomes. The number of mitolysosomes was an average of 6 in the control group, whereas no red puncta were seen in QA-treated BV2 cells (Fig. 1C). Furthermore, confocal imaging of HMC3 cells stained with MitoTracker Deep Red revealed a higher percentage of fused and intermediate mitochondria in QA treated HMC3 cells after 72 h treatment (Additional file 1: Fig. S1C). In contrast, mitochondria in control cells displayed a higher percentage of fragmented mitochondria, which is consistent with the fact that mitochondrial fission plays an essential role in mitophagy (Additional file 1: Fig. S1C). Taken together, these results suggest that QA impairs mitophagy by inhibiting mitolysosome formation in microglial cells.

We next investigated whether UA, a potent mitophagy-inducing agent, restores QA-impaired mitolysosome formation in HMC3 cells transfected with Cox8-EGFP-mCherry construct. UA significantly increased the total number of mitolysosomes in QA-treated HMC3 cells compared to QA-alone treated cells at 24 h (Fig. 1D, E). Quantitatively, UA remarkably increased mitolysosome number to an average of 55 per cell in QA-treated HMC3 cells comparable to the control group (Fig. 1E). UA-only treated cells showed a slight increase in overall mitolysosome number compared to the control group, which is found to be statistically insignificant (Fig. 1E). To further validate that the beneficial effect of UA is dependent on mitolysosome formation via mitophagosome and lysosome fusion, we used autophagosome and lysosome fusion inhibitor CQ in QA treated HMC3 cells transfected with Cox8-EGFP-mCherry construct in the absence or presence of UA. The increase in mitolysosomes per cell by UA in QA-treated HMC3 cells was entirely suppressed in the presence of 20 µM CQ (Fig. 1F, G). Thus, these results confirm that UA restores mitophagy impaired by QA by promoting mitolysosome formation via mitophagosome-lysosome fusion in microglial cells.

### Impaired mitolysosome formation causes the accumulation of damaged mitochondria in microglia

**Fig. 2:**
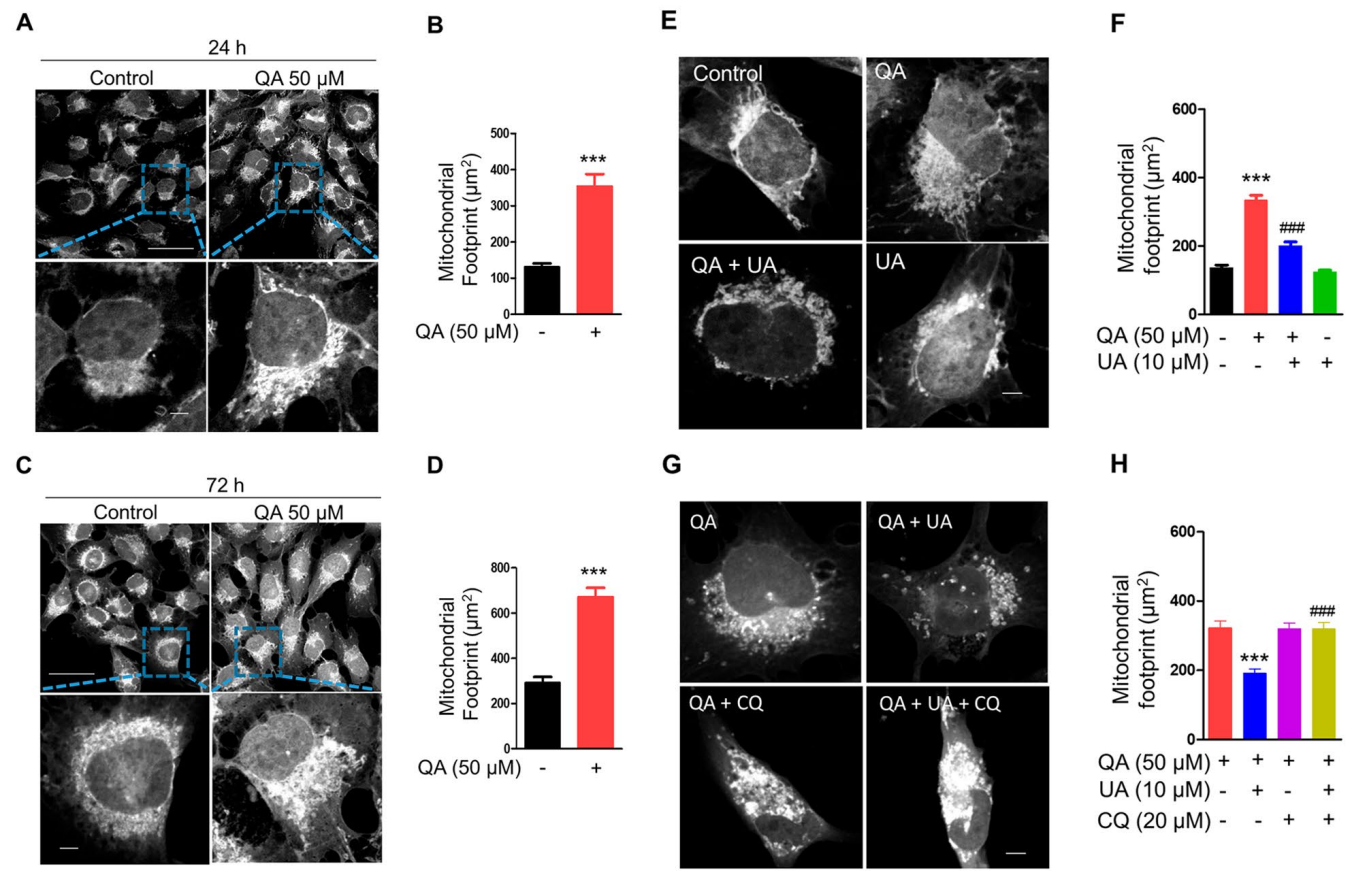
Inhibition of mitolysosome formation by QA accumulates damaged mitochondria in microglia. (**A**, **C**) Representative confocal images of HMC3 cells stained with MitoTracker Deep Red treated with or without 50 µM QA for the indicated time. Insets show higher magnification of the image. Scale bar, 50 μm and 5 μm (ZOOM). (**E**) Representative confocal images of HMC3 cells stained with MitoTracker Deep Red treated with or without 50 µM QA in the absence or presence of 10 µM UA for 24 h. Scale bar, 5 μm. (**G**) Representative confocal images of 50 µM QA-treated HMC3 cells stained with MitoTracker Deep Red in the absence or presence of 10 µM UA and/or 20 µM CQ for 24 h. Scale bar, 5 μm. (**B**, **D**, **F** and **H**) Graphs represent the mitochondrial footprint, which refers to the total area of mitochondria stained with MitoTracker Deep Red (*n* = 15–21 cells). Data are means ± SEM. *** *p* < 0.001 versus no treatment (control) group (**B**, **D** and **F**), *** *p* < 0.001 versus QA alone treatment group (**H**), ^###^*p* < 0.001 versus QA alone treatment group (**F**), ^###^*p* < 0.001 versus QA and UA treatment group (**H**); Two-tailed unpaired *t* test (**B**, **D**) or one-way ANOVA followed by Tukey’s multiple comparisons test (**F** and **H**)

The disruption of mitochondrial homeostasis is considered to play a crucial role in aging and age-associated diseases [33]. As mitophagy is required for maintaining mitochondrial homeostasis [21], we hypothesised that mitophagy impairment by QA could result in less mitochondrial turnover disrupting mitochondrial homeostasis. Consistent with this idea, we found that QA treatment results in a significantly greater percentage of mitochondria inside the HMC3 cells as compared to the control group. Mitochondrial content was increased by approximately 2.7 fold and 2.3 fold following 24 h (Fig. 2A, B) and 72 h (Fig. 2C, D) of QA treatment compared to the control group, respectively. Next, we ruled out mitochondrial biogenesis as a potential cause for the greater mitochondrial content induced by QA. We found that QA had no effect on PGC-1α, a critical co-transcriptional regulator for mitochondrial biogenesis, as measured by western blot (Additional file 1: Fig. S2A, B). Thus, these results suggest that QA-impaired mitochondrial turnover results in the accumulation of mitochondria in microglial cells.

We next analysed whether QA alters mitochondrial function in microglial cells. We found that mitochondrial function was impaired in QA-treated BV2 microglial cells. Loss of mitochondrial function upon QA treatment was confirmed by low MMP, as measured by TMRE fluorescence intensity using flow cytometry, at 24 h and 72 h treatment in a time-dependent manner compared to the control group determined by flow cytometry (Additional file 1: Fig. S3A). To examine the functional consequence of mitochondrial dysfunction, ROS production was measured. Cellular ROS levels increased by approximately 50% in QA-treated BV2 microglial cells at 72 h compared to the control group, as measured with the ROS-sensitive fluorescent probe DCFDA using flow cytometry (Additional file 1: Fig. S3B, C). Together, our data suggest that QA leads to the accumulation of damaged mitochondria in microglial cells.

Furthermore, we investigated whether restoration of mitolysosome formation by UA prevents the accumulation of dysfunctional mitochondrial content in QA-treated HMC3 cells. As expected, compared with QA alone, we found that UA lowered mitochondrial content in HMC3 cells exposed to QA (Fig. 2E, F). Mitochondrial content was lower by ~ 20% following 24 h of UA treatment in QA-treated HMC3 cells compared with QA-alone treated cells (Fig. 2F). We then examined the effects of CQ on UA-induced mitochondrial turnover in QA-treated HMC3 cells. CQ completely abolished the beneficial effects of UA (Fig. 2G, H). Notably, the effect of UA on mitochondrial turnover resulted from the induction of mitophagosome-lysosome fusion. Altogether, these findings suggest that inhibition of mitolysosome formation by QA accumulates damaged mitochondria in microglial cells.

### Impaired mitolysosome formation promotes microglial senescence

**Fig. 3:**
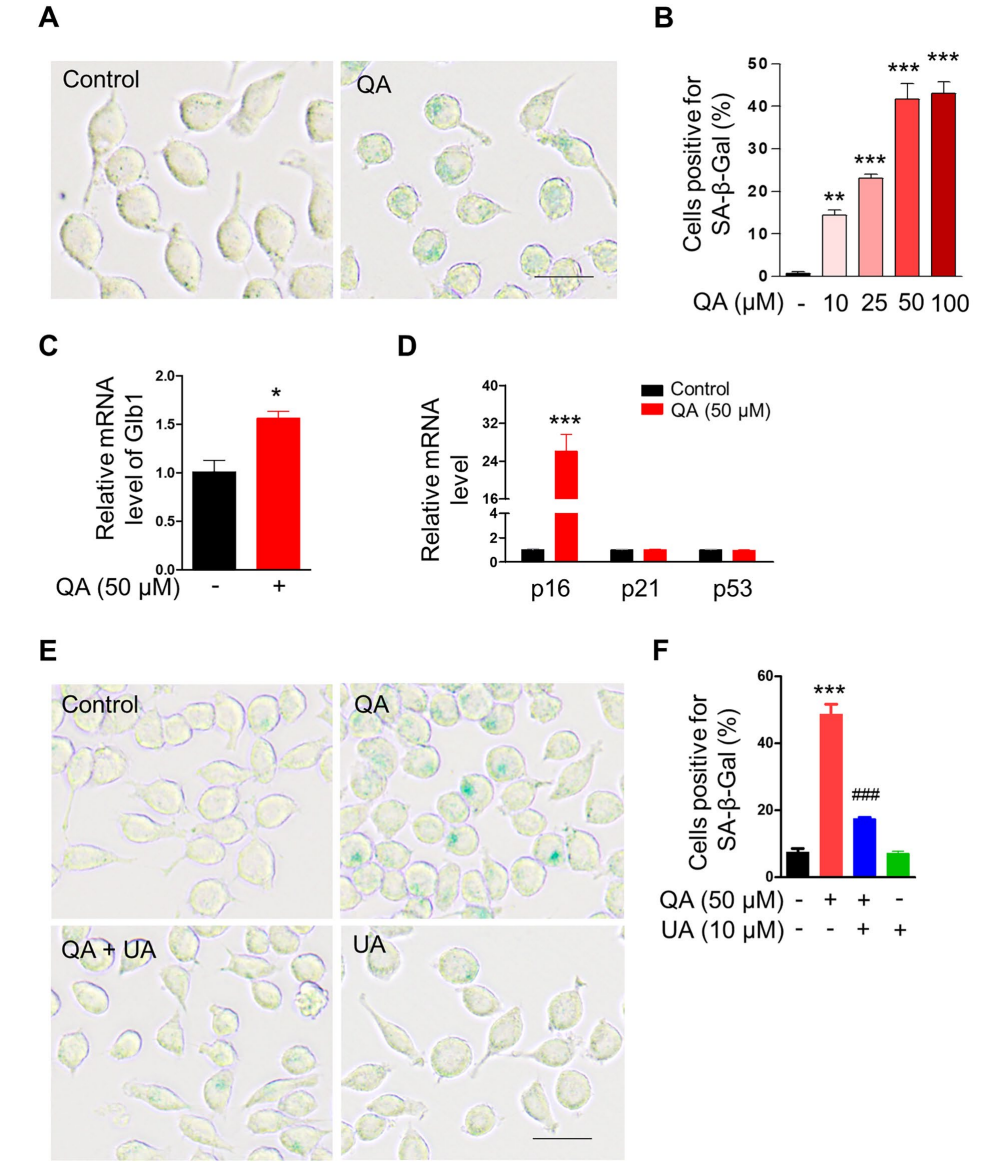
Inhibition of mitolysosome formation by QA induces microglial senescence. (**A**) Representative images of BV2 cells stained with SA-β-Gal treated with or without 50 µM QA for 72 h. Scale Bar, 50 μm. (**C**) Relative mRNA level of Glb1 in BV2 cells treated with or without 50 µM QA for 72 h (*n* = 3). (**D**) Relative mRNA levels of p16, p21 and p53 in BV2 cells treated with or without 50 µM QA for 72 h (*n* = 2–4). (**E**) Representative images of BV2 cells stained with SA-β-Gal treated with or without 50 µM QA in the absence or presence of 10 µM UA for 72 h. Scale Bar, 50 μm. (**B, F**) Graphs represent percent of SA-β-Gal positive cells (green staining) per imaging field. Data are means ± SEM (**B**) *n* = 5–6 imaging fields and (**F**) *n* = 3 imaging fields; * *p* < 0.05, ** *p* < 0.01 and *** *p* < 0.001 versus no treatment (control) group, ^###^*p* < 0.001 versus QA alone treatment group; One-way ANOVA followed by Tukey’s multiple comparisons test (**B** and **F**) or two-tailed unpaired *t* test (**C**, **D**)

Accumulation of damaged mitochondria is a hallmark of aging and age-related diseases [33]. Since our results demonstrate that QA led to the accumulation of dysfunctional mitochondria in microglial cells, we further ask whether QA induces microglial senescence, an important driver of aging, in BV2 microglia. When BV2 cells were treated with a range of doses of QA between 10 and 100 µM for 72 h, elevated SA-β-Gal activity was observed compared to the control group in a dose-dependent manner (Fig. 3A, B). Furthermore, qRT-PCR revealed approximately 1.6 fold increase in Glb1 mRNA levels in QA-treated BV2 cells compared to the control group after 72 h of treatment (Fig. 3C), confirming accelerated senescent phenotype in QA-treated BV2 cells. We then analysed the effects of QA on cell cycle inhibitors p16, p21 and p53 using qRT-PCR. We found approximately 26 fold increase in p16 mRNA levels in QA-treated BV2 cells compared to the control group after 72 h of treatment but not p21 and p53 mRNA levels (Fig. 3D). Furthermore, to test whether QA-inhibited mitolysosome formation is associated with accelerated senescent phenotype, we treated BV2 cells with 10 µM UA for 72 h in the absence or presence of QA. UA significantly reduced the increased SA-β-Gal activity by approximately 64% in QA-treated BV2 cells compared to QA-alone treated cells (Fig. 3E, F). Thus, our results suggest that the microglial senescence induced by QA could be due to the inhibition of mitolysosome formation by QA in microglial cells.

### Impaired mitolysosome formation leads to poor healthspan in *C. elegans*

**Fig. 4:**
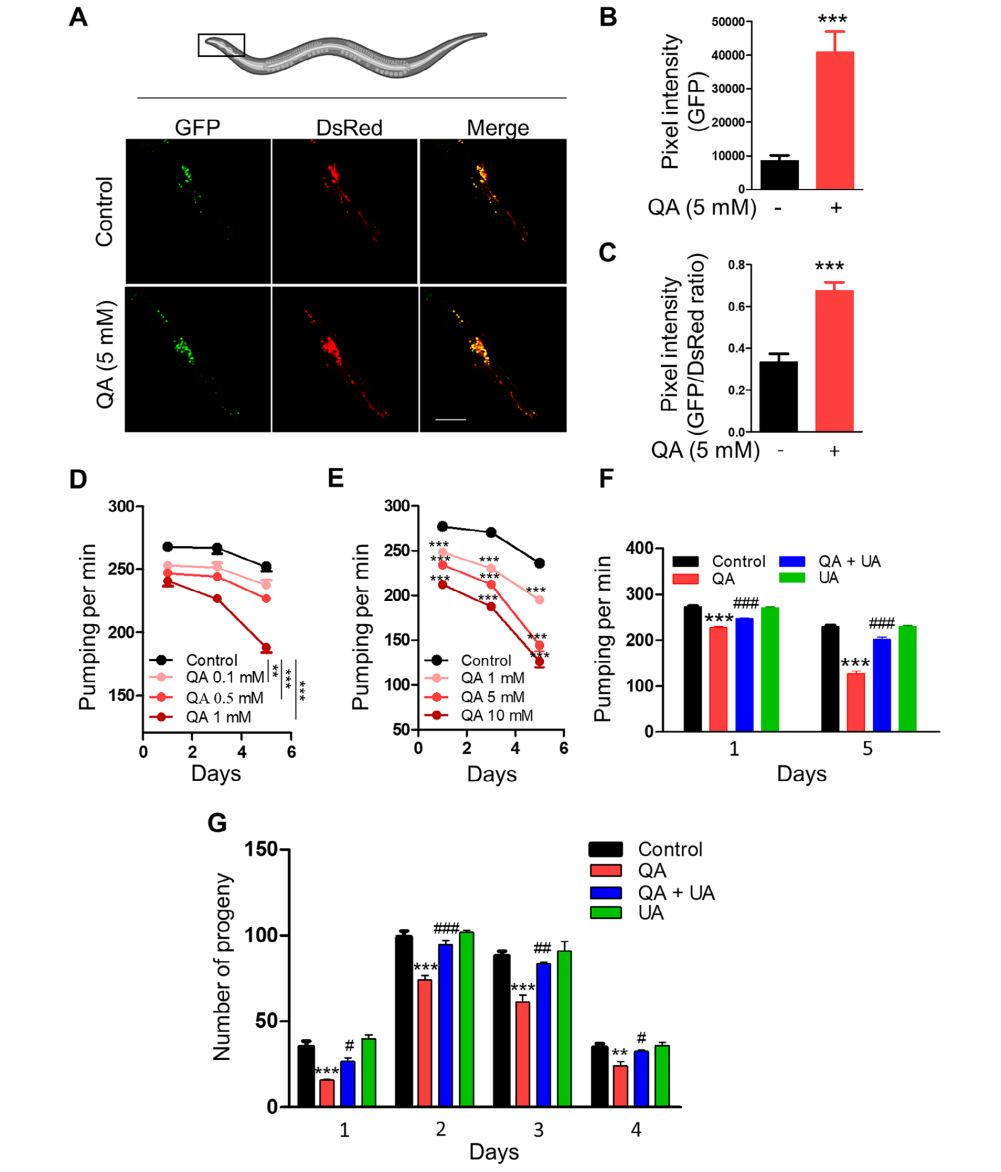
Inhibition of mitolysosome formation by QA induces poor healthspan in *C. elegans.* (**A**) Representative images showing the level of mitophagy in anterior head neurons at day 5 of adulthood in worms expressing mt-Rosella reporter treated with or without 5 mM QA. Scale bar, 50 μm. (**B**) Relative levels of neuronal mitophagosome are expressed as the GFP fluorescence intensity (*n* = 6 worms). (**C**) Relative levels of neuronal mitolysosomes are expressed as the ratio of GFP fluorescence intensity and DsRed fluorescence intensity (*n* = 6–8 worms). (**D, E**) Pharyngeal pumping at days 1, 3 and 5 of adulthood in worms treated with or without QA as indicated (**D**) *n* = 9 worms, (**E**) *n* = 13 worms. (**F**) Pharyngeal pumping at days 1 and 5 of adulthood in worms treated with or without 5 mM QA in the absence or presence of 50 µM UA (*n* > 11 worms). (**G**) Number of progeny at days 1, 2, 3 and 4 of adulthood in worms treated with or without 5 mM QA in the absence or presence of 50 µM UA (*n* = 5 worms). Data are mean ± SEM; ** *p* < 0.01 and *** *p* < 0.001 versus no treatment (control) group, ^#^*p* < 0.05, ^##^*p* < 0.01 and ^###^*p* < 0.001 versus QA alone treatment group; Two-tailed unpaired *t* test (**B, C**) or two-way repeated measures ANOVA, followed by Bonferroni post-tests (**D** and **E**) or One-way ANOVA followed by Tukey’s multiple comparisons test (**F** and **G**)

To further investigate whether QA impairs mitolysosome formation in vivo, we used transgenic nematodes with a pan-neuronal system expressing mitochondria-targeted Rosella [32]. Here we found that QA (5 mM) increased both the GFP fluorescence intensity and the ratio of GFP to DsRed fluorescence intensity compared to controls in 5-day-old adult worms, indicating that QA promotes neuronal mitophagosome formation but impairs mitolysosome formation in *C. elegans* (Fig. 4A-C). To understand the role of QA on aging in *C. elegans*, we next evaluated the healthspan associated with aging by assessing pharyngeal pumping rate and fertility in *C. elegans*. QA dose ranges between 0.1 mM and 10 mM dramatically reduced pumping rate in worms in a dose-dependent manner over time compared to control worms, with 5 mM showing approximately 39% reduction at day 5 of adulthood (Fig. 4D, E). We then investigated whether inhibition of mitolysosome formation by QA contributes to reduced pharyngeal pumping rate in worms. UA significantly improves the pharyngeal pumping rate in worms treated with 5 mM QA compared to worms treated with 5 mM QA alone at day 1 and day 5 of adulthood (Fig. 4F). UA alone did not appear to alter pharyngeal pumping compared to the control worms (Fig. 4F). Furthermore, worms treated with 5 mM QA exhibited a significant reduction in their number of progeny compared to control worms from day 1 to day 4 of adulthood (Fig. 4G). On the other hand, UA significantly prevents the reduction in the number of progeny in worms treated with 5 mM QA compared to worms treated with 5 mM QA alone from day 1 to day 4 of adulthood (Fig. 4G). Taken together, these results suggest that the compromised mitophagy induced by QA may accelerate poor healthspan in *C. elegans*, and UA is able to prevent these alterations.

### Oxidative stress impairs p62 degradation in microglia

**Fig. 5:**
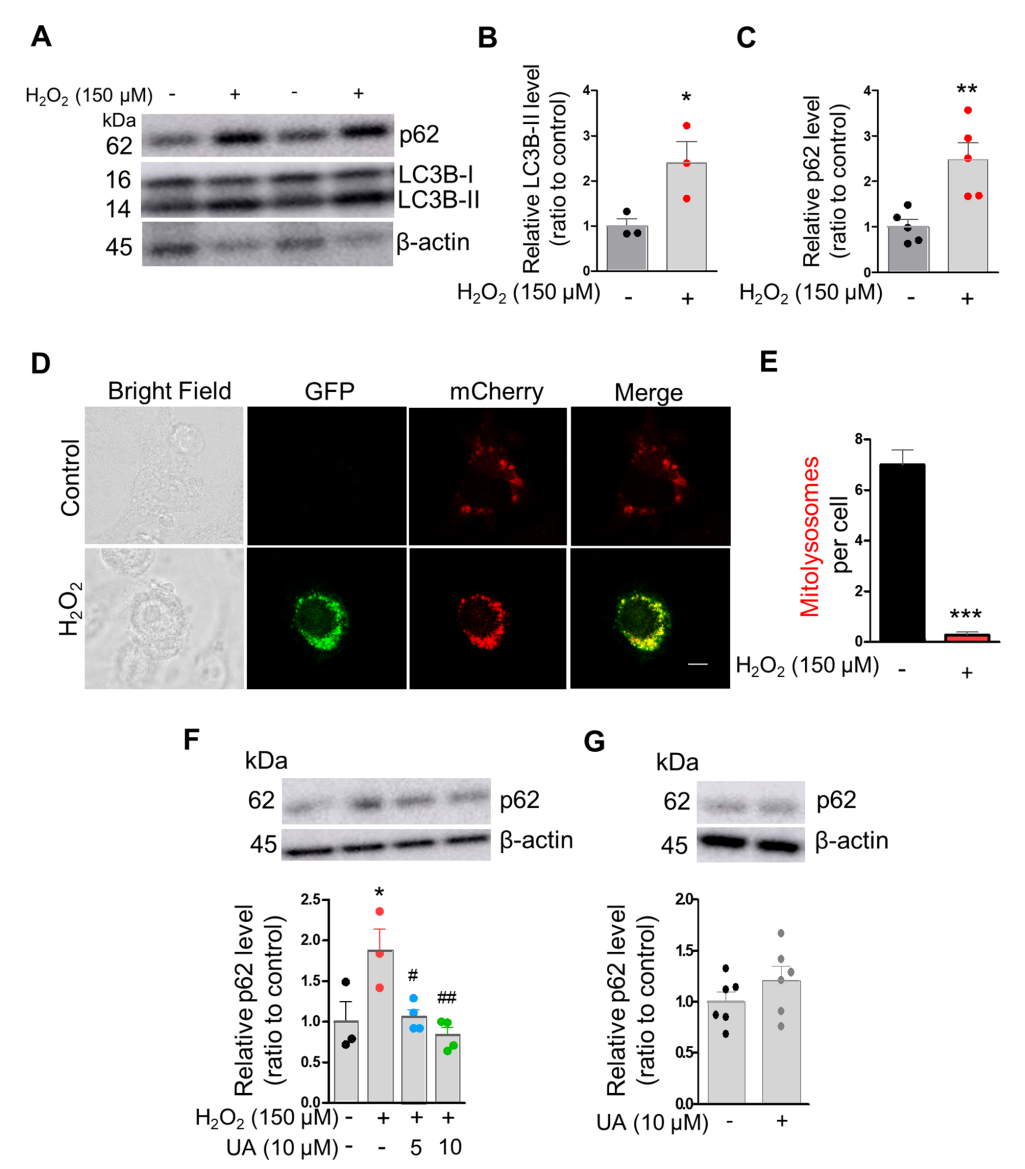
Oxidative stress impairs p62 degradation in microglia. (**A**) Representative western blot images of LC3B-I/II and p62 in BV2 microglia treated with or without 150 µM H_2_O_2_ for 72 h. β-actin was used for loading control. (**B**) Quantification of relative protein level of LC3B-II in **A (***n* = 3). (**C**) Quantification of relative protein level of p62 in **A** (*n* = 5). (**D**) Representative confocal images of BV2 cells transiently transfected with Cox8-EGFP-mCherry plasmid with or without 150 µM H_2_O_2_ treatment for 72 h. Scale bar, 5 μm. (**E**) Graph represents the number of mitolysosomes (red dots) per cell in **D** (*n* = 18 cells). (**F**) Representative western blot image and quantification of relative protein level of p62 in BV2 microglia treated with or without 150 µM H_2_O_2_ in the absence or presence of UA as indicated for 72 h. β-actin was used for loading control (*n* = 3–4). (**G**) Representative western blot image and quantification of relative protein level of p62 in BV2 microglia treated with or without 10 µM UA for 72 h. β-actin was used for loading control (*n* = 6). Data are means ± SEM. *p < 0.05, **P < 0.01 and ***P < 0.001 versus no treatment (control) group, ^#^*p* < 0.05 and ^##^*p* < 0.01 versus H_2_O_2_ alone treatment group; Two-tailed unpaired *t* test (**B**, **C**, **E** and **G**) or one-way ANOVA followed by Tukey’s multiple comparisons test (**F**)

Oxidative stress is one condition that induces impairment of autophagic flux [34]. Indeed, we observed increased production of cellular ROS by QA in microglial cells. We next evaluated whether oxidative stress impairs mitophagy in microglial cells. We used H_2_O_2_ to induce oxidative stress damage in microglial cells and analysed the protein level of general autophagy markers LC3B-II and p62 by western blot. H_2_O_2_ treatment significantly increased the formation of autophagosomes, as indicated by increased expression of LC3B-II proteins compared to the control group after 72 h of treatment (Fig. 5A, B). However, H_2_O_2_ treatment prevents p62 degradation, as demonstrated by the increased p62 protein level in H_2_O_2_-treated cells compared to the control group after 72 h of treatment (Fig. 5A, C). To confirm whether H_2_O_2_-impaired p62 degradation is related to impaired mitolysosome formation, we examined mitolysosomes in BV2 cells expressing Cox8-EGFP-mCherry. Consistent with QA, we found decreased mitolysosomes in H_2_O_2_-treated cells at 72 h compared to the control group (Fig. 5D, E). In contrast, UA (5 µM and 10 µM) significantly decreased p62 levels in a dose-dependent manner in H_2_O_2_-treated BV2 cells compared to H_2_O_2_ alone treated cells (Fig. 5F). However, UA (10 µM) alone treated BV2 cells showed comparable p62 levels to the control group (Fig. 5G), suggesting that the lower level of p62 by UA in H_2_O_2_-treated cells were caused by mitophagy elevation rather than decreased protein expression by UA. Thus, these results demonstrate that oxidative stress impairs mitophagic activity by inhibiting mitolysosome formation in microglial cells consistent with QA.

### Excessive oxidative stress causes mitochondrial dysfunction in microglia

Considering that defective mitophagy results in the accumulation of dysfunctional mitochondria, we next assessed whether H_2_O_2_ influences the function of mitochondria. We estimated MMP using TMRE to demonstrate mitochondrial function. Indeed, we found that MMP was reduced by H_2_O_2_ treatment compared to control in BV2 microglial cells at 72 h (Additional file 1: Fig. S4A, B). To further ascertain the effects of H_2_O_2_ on mitochondria, we measured mitochondrial ROS levels using MitoSOX staining. Accordingly, H_2_O_2_ substantially increased the production of mitochondrial ROS levels in BV2 cells compared to control at 72 h (Additional file 1: Fig. S4C, D). Thus, these results suggest that H_2_O_2_ treatment causes mitochondrial dysfunction in microglial cells. Furthermore, we examined the effects of UA on the function of mitochondria after H_2_O_2_ treatment. Our results showed that 5 µM and 10 µM UA further decreased MMP in a dose-dependent manner in H_2_O_2_-treated BV2 cells compared to H_2_O_2_ alone treated cells (Additional file 1: Fig. S4E, F), indicating increased mitochondrial depolarisation by UA in order to facilitate mitophagy.

### Oxidative stress can impair mitophagy causing microglial senescence

**Fig. 6:**
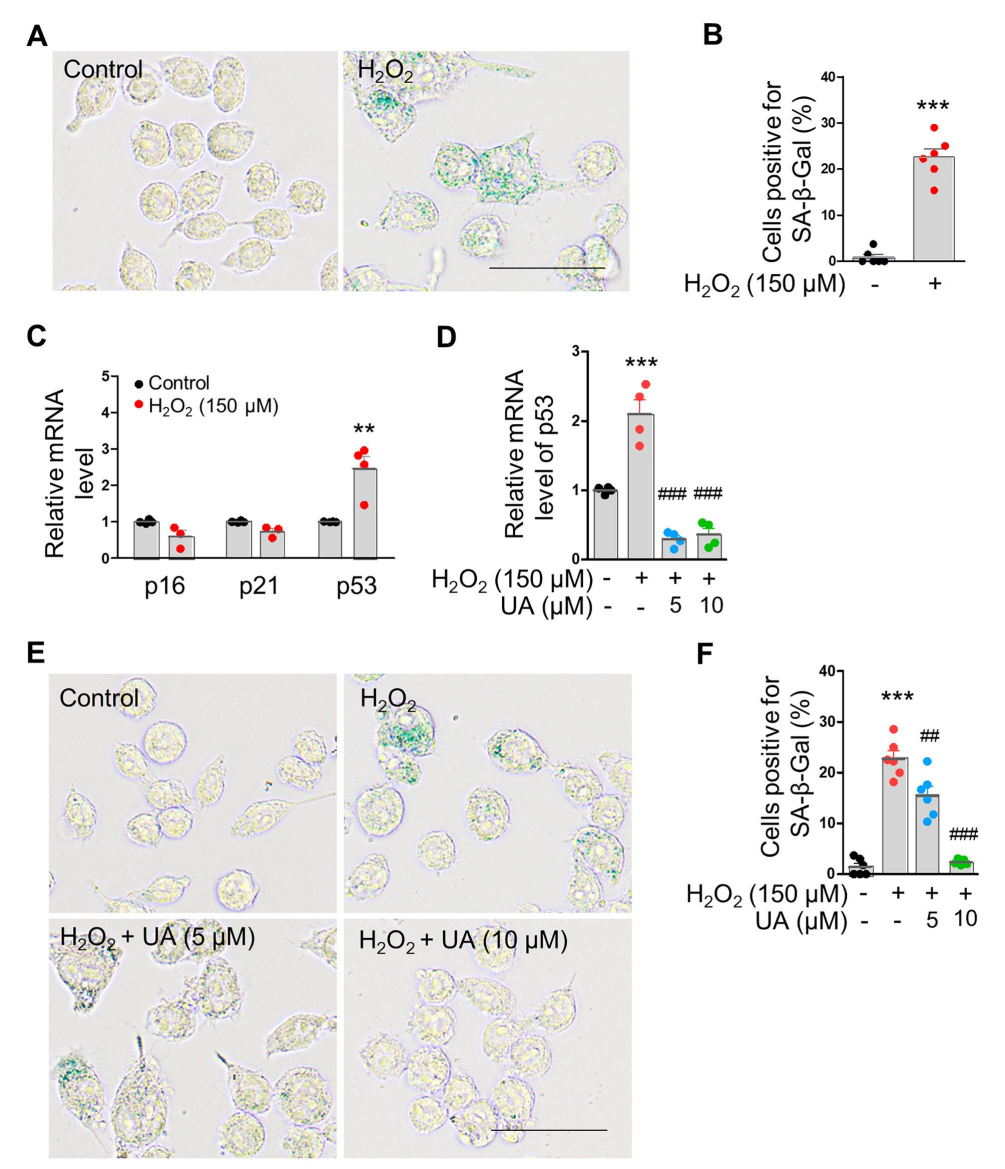
Inhibition of mitolysosome formation by oxidative stress induces microglial senescence. (**A**) Representative images of BV2 cells stained with SA-β-Gal treated with or without 150 µM H_2_O_2_ for 72 h. Scale Bar, 50 μm. (**C**) Relative mRNA levels of p16, p21 and p53 in BV2 cells treated with or without 150 µM H_2_O_2_ for 72 h (*n* = 3–4). (**D**) Relative mRNA level of p53 in BV2 cells treated with or without 150 µM H_2_O_2_ in the absence or presence of UA as indicated for 72 h (*n* = 4). (**E**) Representative images of BV2 cells stained with SA-β-Gal treated with or without 150 µM H_2_O_2_ in the absence or presence of UA as indicated for 72 h. Scale Bar, 50 μm. (**B, F**) Graphs represent percent of SA-β-Gal positive cells (green staining) per imaging field. (**B**) *n* = 6 imaging fields and (**F**) *n* = 5–6 imaging fields. Data are means ± SEM; ** *p* < 0.01 and *** *p* < 0.001 versus no treatment (control) group, ^##^*p* < 0.01 and ^###^*p* < 0.001 versus H_2_O_2_ alone treatment group; Two-tailed unpaired *t* test (**B** and **C**) or one-way ANOVA followed by Tukey’s multiple comparisons test (**D** and **F**)

Increased ROS and oxidative stress are widely implicated in aging and age-related diseases [35]. Here, we investigated whether H_2_O_2_ can induce cellular senescence in microglial cells using senescence markers SA-β-Gal and the cell cycle inhibitors p16, p21 and p53. Microglia treated with 150 µM H_2_O_2_ exhibited a significant increase in SA-β-Gal activity at 72 h of treatment (Fig. 6A, B). Following the application of 150 µM H_2_O_2_, the percentage of SA-β-Gal-positive cells was 22 times higher than the control group (Fig. 6B). We further analysed the mRNA level of p16, p21 and p53 with 150 µM H_2_O_2_ exposure. qRT-PCR revealed 2.5 fold increase in p53 mRNA levels compared to the control group after 72 h of treatment but not p16 and p21 mRNA levels (Fig. 6C). Thus, the increase in the senescence markers SA-β-Gal and p53 demonstrate the potential of oxidative stress to induce senescence in microglia. We further investigated whether H_2_O_2_-impaired mitolysosome formation could play a significant role in inducing microglial senescence by H_2_O_2_. Accordingly, 5 µM and 10 µM UA-treated cells displayed a significant decrease in SA-β-Gal activity by approximately 32% and 90% in H_2_O_2_-treated BV2 cells at 72 h compared to H_2_O_2_ alone treated cells, respectively (Fig. 6E, F). Further, UA at 5 µM and 10 µM doses significantly reduced the increased p53 mRNA level in H_2_O_2_-treated cells compared to H_2_O_2_ alone treated cells at 72 h of treatment (Fig. 6D). Taken together, these results suggest that mitophagy impairment via inhibition of mitolysosome formation is crucial for oxidative stress-induced senescence in microglial cells.

In addition to chronic stress, inflammation and metabolic condition, Aβ, a pathological hallmark of age-related AD, may induce QA overproduction via microglia activation. We tested the effect of Aβ on QA production in BV2 microglia with or without primary hippocampal neurons. Here, we found that Aβ_1−42_ stimulation increased QA concentration by approximately 28% in BV2 cells at 48 h treatment compared to the control cells, as measured by HPLC (Additional file 1: Fig. S5A). Interestingly, we found a 37% increase in the concentration of QA in microglial co-culture with primary hippocampal neurons at 48 h treatment with Aβ_1−42_ compared with untreated microglial and primary hippocampal neuronal co-culture (Additional file 1: Fig. S5B). Indeed, primary hippocampal neurons did not produce QA with or without Aβ_1−4_ treatment in the absence of microglial cells (Additional file 1: Fig. S5B). To further confirm the increased production of QA, we detected the protein level of 3-HAAO enzyme by western blot. Consistently, Aβ_1−42_ resulted in an approximately 3.3 fold increase in 3-HAAO protein expression in microglia and primary hippocampal neurons co-culture compared to the control cells at 48 h treatment (Additional file 1: Fig. S5C, D). The increased amount of QA by Aβ_1−42_ treated microglia co-cultured with primary hippocampal neurons was prevented by 10 µM Ro 61-8048, the inhibitor of kynurenine 3-hydroxylase enzyme involved in the kynurenine pathway, at 48 h treatment (Additional file 1: Fig. S5F). The basal level of QA concentration produced by microglia in the absence or presence of primary hippocampal neurons was also significantly reduced by Ro 61-8048 at 48 h treatment (Additional file 1: Fig. S5E, F). Thus, these results suggest that QA levels elevate in response to microglial activation in the brain, which may contribute to the progression of aging and age-related diseases.

## Discussion

QA is a cytotoxic metabolite of the kynurenine pathway, which plays a crucial role in neuronal damage [36]. Brain aging and age-related neurodegenerative diseases, including AD, are associated with increased concentrations of QA [11–13]. Our study, for the first time, revealed that increased QA impairs mitophagy by inhibiting the formation of mitolysosomes in microglial cells and in the neurons of *C. elegans*. Further, we show that mitophagy impairment by QA leads to the accumulation of damaged mitochondria, thereby promoting age-related phenotypes such as microglial senescence and poor healthspan in *C. elegans*. Moreover, we show that oxidative stress is a potential mediator of QA-induced mitophagy impairment and age-related phenotype in microglia. Finally, our results show that restoration of mitophagy by UA prevents the accumulation of damaged mitochondria and rescues age-related phenotypes induced by QA in both microglia and *C. elegans*.

Senescent microglia are a distinct microglial phenotype in the aging brain that has been implicated in the progression of aging and age-related neurodegenerative diseases [3, 4, 7]. Our study, for the first time, demonstrated that an increased QA induces microglial senescence. Furthermore, we used *C. elegans* to study the role of QA on age-associated healthspan. We showed that QA accelerated age-associated decline of pumping rate and fertility, indicative of an accelerated aging process. These results support our previously reported idea that an increased QA accelerates the aging process, although the immune cells were not investigated [14]. Therefore, microglial senescence induced by QA attributes an important part of brain aging. Unravelling how QA causes microglial aging is of utmost importance.

Mitophagy is a cellular quality control system that is responsible for the clearance of damaged mitochondria [21]. Here, we provide evidence for the first time that QA impairs mitophagy in microglial cells and in the anterior head neurons of *C. elegans*. Further, we demonstrate that mitophagy impairment by QA results in the accumulation of mitochondria. Here we also showed that the inhibitory effect of QA on mitophagy was not due to the blockade in the initiation of mitophagy, as QA increased the expression of LC3B-II protein, a marker for autophagosomes. Instead, we revealed that QA inhibits mitophagy, specifically at the mitolysosome formation stage, as there was a reduction in the formation of mitolysosomes in both microglial cells and in the anterior head neurons of *C. elegans*. Thus, these results provide new insights into the mechanisms by which QA affects mitophagy. Additionally, the impairment of mitolysosome formation by QA could be due to an impaired mitophagosome and lysosomal fusion associated with the alteration in SNARE proteins. SNARE proteins are the proteins involved in the fusion of autophagosomes and lysosomes. SNARE proteins have been implicated in the development of age-related neurodegenerative diseases [37]. However, further experiments are required to determine if QA regulates SNARE proteins and causes the impairment of mitolysosome formation.

Our study indicates that QA-induced mitochondrial mass is primarily due to limited mitochondrial turnover caused by impaired mitophagy. The involvement of mitochondrial biogenesis was unlikely, as there was no difference in the level of the PGC-1α in microglial cells stimulated with QA. In parallel, we showed that QA increased mitochondrial damage by demonstrating a reduction in MMP and excessive cellular ROS production, suggesting the accumulation of damaged mitochondria rather than healthy mitochondria. These results are consistent with the previous studies that have reported alterations in mitochondrial function in rats after treatment with QA [17, 18]. Together, our results suggest that impaired mitolysosome formation caused by QA leads to a decrease in mitochondrial turnover and, therefore accumulation of damaged mitochondria.

Of note, defective mitophagy and the accumulation of dysfunctional mitochondria are associated with senescence or age-related diseases [23, 25]. Indeed, we observed that the impairment of mitophagy induced by QA was involved in establishing the senescent phenotype in response to chronic QA stimulation in microglia. Our results demonstrate that restoring mitophagy by UA prevented the accumulation of damaged mitochondria and the establishment of senescence in microglial cells. Furthermore, we show that the stimulation of mitophagy by UA improved the age-associated decline in healthspan accelerated by QA in *C. elegans*. Thus, our results suggest impaired mitophagy mediates neuroinflammation-induced aging and age-related phenotypes, potentially accelerating age-related diseases as demonstrated for stress-driven senescence [25, 26].

Over production of ROS is a key factor that plays an important role in aging and age-related diseases [35]. Previously, oxidative stress has been shown to contribute to cell senescence in different cell types [34, 38, 39]. In our study, stimulation with QA increased cellular ROS in microglial cells. Here, we demonstrated that H_2_O_2_ treatment can recapitulate the features of senescence similar to QA in microglia. Therefore, it is likely that the cellular senescence after QA administration is via excess ROS production. In agreement with this, oxidative stress has been demonstrated to play a significant role in the hippocampal damage induced by QA in vivo [40]. Further, we found that H_2_O_2_ treatment phenocopies the blockage of mitophagy caused by QA stimulation with the accumulation of LC3B-II and p62 levels. This is further supported by the reduction in mitolysosome formation in H_2_O_2_-stimulated microglial cells consistent with QA stimulation. Additionally, the mitochondrial damage by H_2_O_2_ was evidenced by decreased MMP and increased mitochondrial ROS accumulation in microglia, further exacerbating cellular ROS levels in microglia in a vicious cycle. Recently, autophagosome and lysosome fusion blockage has been found to promote cell toxicity via excess ROS production [41]. Therefore, our results indicate oxidative stress as a mediator of QA-induced senescence in microglia, as already demonstrated for stress-driven senescence in non-microglial cells [25, 42].

UA is a natural compound produced by gut bacteria from ingested ellagitannins and ellagic acid, complex polyphenols abundant in foods such as pomegranate, berries, and nuts [43]. Previous studies have shown the capacity of UA to stimulate mitophagy in various biological systems, including cell cultures, nematodes, microglia and neurons in mice [22, 23, 44]. Our study demonstrates that UA enhances mitophagy and mitochondrial turnover impaired by QA in microglial cells. More importantly, our results provide insight into the molecular mechanism by which UA promotes mitophagy against QA toxicity. Interestingly, we found that UA was effective in promoting the fusion between mitophagosome and lysosome because CQ, a well-known inhibitor of autophagosome and lysosome fusion [45], prevented mitophagy restoration by UA. Also, we showed that the restoration of mitochondrial turnover by UA was completely suppressed in the presence of CQ. Thus, our study suggests that UA regulates mitophagy via mitophagosome-lysosome fusion. Furthermore, we found that UA prevents cellular senescence induced by QA in microglial cells and improves age-related healthspan in QA-treated

*C. elegans*, suggesting a potential of UA to protect against cellular aging and age-related conditions by maintaining mitochondrial homeostasis through mitophagy induction. This is in line with several preclinical studies that show that UA protects against cellular aging and age-related diseases by increasing mitophagy, mitochondrial function and reducing detrimental inflammation [22, 23, 46, 47]. Although a previous study reported that UA does not have typical anti-oxidant properties [22], we found that UA prevents H_2_O_2_-induced impaired mitophagy and microglial senescence. Our study suggests that the anti-aging potential of UA may lie in improving mitophagy and, in turn, anti-oxidative and anti-inflammatory effects as well as neuroprotection. Therefore, the current study verifies that UA was able to restore mitophagy in both microglia and neurons. The broader impacts of UA in targeting multiple aspects, such as glial dysfunction and neuronal death, support the potential of UA in maintaining an interconnected network between neurons and microglia to maintain brain homeostasis during aging. Given the importance of mitophagy impairment by QA in the progression of aging, restoration of mitophagy by UA in both neuronal and microglial cells may be a promising anti-aging therapeutic approach for the prevention and treatment of neuroinflammation-associated with increased QA in brain aging diseases. Nonetheless, the limitations of our study are [1] to extend this work to rodent studies, [2] in depth investigation of mitophagosome and lysosome fusion including SNARE protein complex, and lysosome integrity and acidic conditions and [3] a complete understanding of the steps between mitophagy and cell senescence.

Finally, our findings consistently show that microglial stimulation with Aβ leads to an increased level of QA, in agreement with previous research that demonstrates increased expression of QA by microglia following stimulation with Aβ [9]. For the first time, this study reported that Aβ stimulates microglia, leading to the subsequent upregulation of the enzyme 3-HAAO. The 3-HAAO enzyme plays a pivotal role in the kynurenine pathway, specifically catalysing the conversion of 3-Hydroxyanthranilic acid to QA. Furthermore, for the first time, we showed that microglia stimulated by Aβ results in a further increase in QA production when co-cultured with primary hippocampal neurons. Previous research on brain injury has shown that microglia are rapidly activated in response to synaptic degeneration and promote the elimination of synaptic material in adult mice [48]. Similarly, another study focusing on the hypothalamus, an area critical for energy homeostasis, demonstrated inflammation induced by a high-fat diet is a manifestation of neuronal injury [49]. Thus, our findings highlight the relevance of Aβ triggered microglial activation followed by QA over production contributing to the neuropathology of neurodegenerative diseases. It is known that QA is increased in a number of neurodegenerative diseases [11, 50, 51]. This study indicates that QA impairs mitophagy flux, which provides a potential new therapeutic target in neurodegenerative diseases. Importantly, we showed that the mitophagy inducer ameliorates cell senescence and prolongs ageing in *C. elegans* while these discoveries need to be confirmed in other animal models and human studies.

## Conclusions

In conclusion, this study indicates that an increased QA impairs mitolysosome formation, which in turn causes immune cell dysfunction and poor healthspan. This pathology may play a central role in neuroinflammation-induced diseases related to aging. Importantly, our study suggests that mitophagy inducers such as UA could be a promising anti-aging candidate to prevent and treat neuroinflammation-induced diseases related to aging.

### Electronic supplementary material

Below is the link to the electronic supplementary material.

**Additional file 1**: Additional figures and tables

**Additional File 2**: Western blots full scans

## Data Availability

The data and materials generated during the current study are not publicly available but are available from the corresponding author upon reasonable request.

## Abbreviations

3-HAAO: 3-Hydroxyanthranilic acid 3, 4-dioxygenase
AD: Alzheimer’s disease
Aβ: Amyloid-β
CQ: Chloroquine
DIV: Days in vitro
HPLC: High-performance liquid chromatographic
NGM: Nematode growth media
H_2_O_2_: Hydrogen peroxide
MMP: Mitochondrial membrane potential
PMSF: Phenylmethanesulfonyl fluoride
QA: Quinolinic acid
ROS: Reactive oxygen species
SA-β-Gal: Senescence associated-β-galactosidase
TMRE: Tetramethylrhodamine ethyl ester
UA: Urolithin A
