# Supplementary material for: Quinolinic acid impairs mitophagy promoting microglia senescence and poor healthspan in C. elegans: a mechanism of impaired aging process
Source: Biol Direct. 2023 Dec 20;18:86. doi: 10.1186/s13062-023-00445-y (PMC10734169; doi:10.1186/s13062-023-00445-y)
Supplement: Supplementary file 1 — Additional file 1: Additional figures and tables [file 13062_2023_445_MOESM1_ESM.docx]

Supplementary materials

**Quinolinic acid impairs mitophagy promoting microglia senescence and poor healthspan in *C. elegans*: A mechanism of impaired aging process**

Anjila Dongol, Xi Chen, Peng Zheng, Zehra Boz Seyhan and Xu-Feng Huang

Supplementary figures and legends


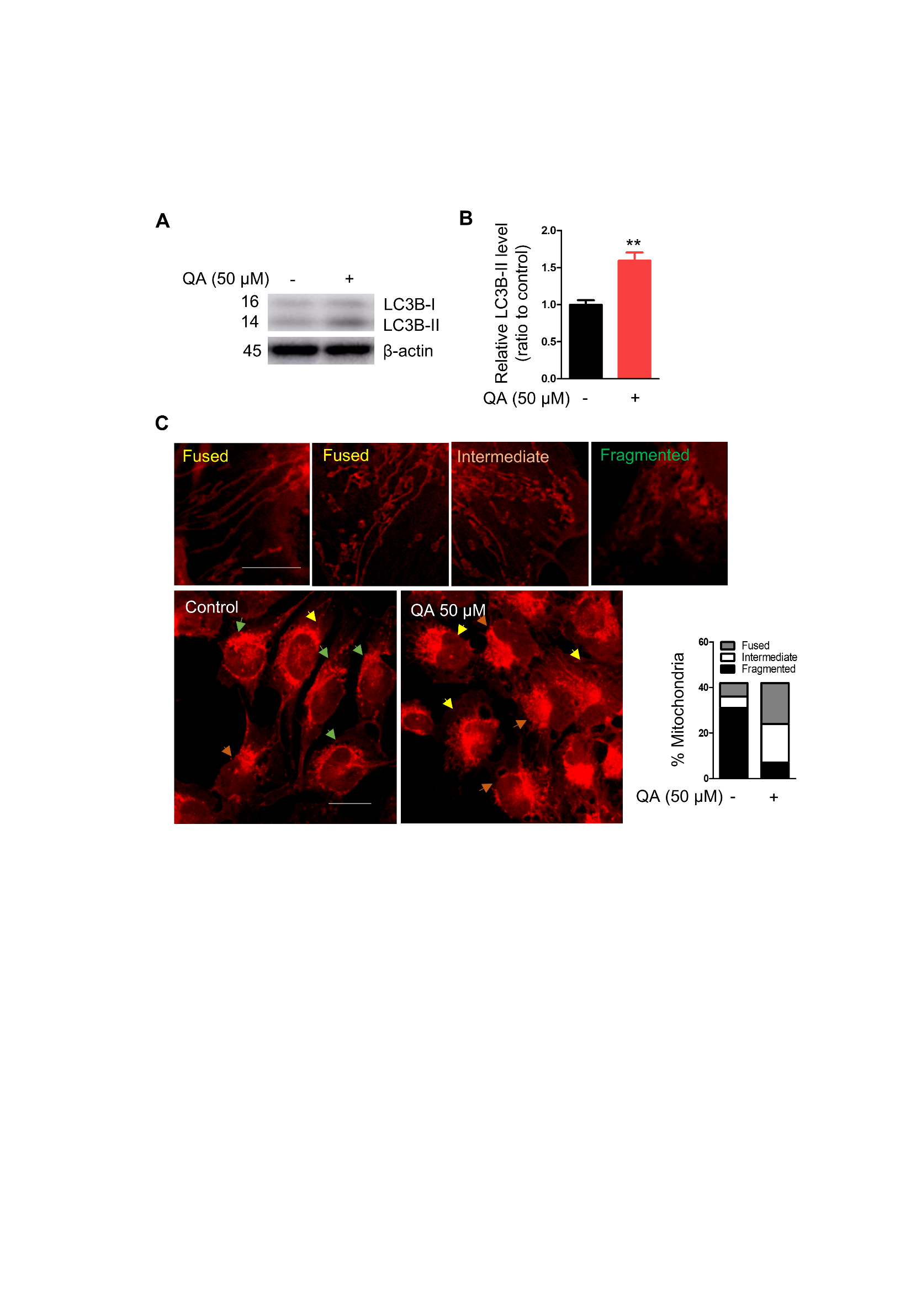


**Fig. S1. QA promotes mitophagosome formation and mitochondrial elongation in microglia.** (**A**) Representative western blot image of LC3B-I/II in BV2 microglia treated with or without 50 µM QA for 72 h. β-actin was used for loading control. (**B**) Quantification of relative protein level of LC3B-II in **A** (*n* = 3). (**C,** Top panel) Representative confocal images showing different types of mitochondrial morphology in HMC3 cells stained with MitoTracker Deep Red. Scale bar, 10 µm. (**C,** Bottom panel) Representative confocal images of HMC3 cells stained with MitoTracker Deep Red treated with or without 50 µM QA for 72 h. Scale bar,
25 µm. Graph represents different types of mitochondrial morphologies observed in cells in (**C,** Bottom panel). Green arrows indicate “Fragmented” mitochondria with complete fragmentation, resulting in only mitochondrial spheres. Orange arrows indicate “Intermediate” mitochondria with extensive fragmentation but contain some very short mitochondrial rods (< 5 μm in length). Yellow arrows indicate “Fused” mitochondria with medium-length mitochondrial tubules (> 5 μm in length), regardless of whether there was accompanying fragmentation or not. Some 42 cells per condition were scored. Data are means ± SEM.
** *p* < 0.01 versus no treatment (control) group; Two-tailed unpaired *t* test (**B**).

**
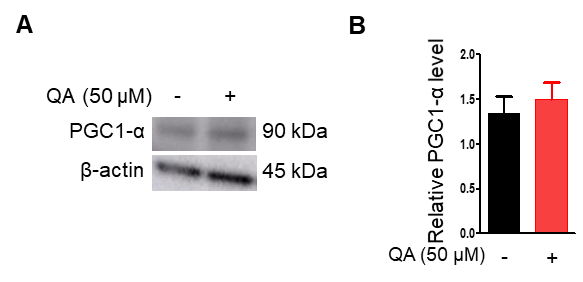
**

**Fig. S2. QA does not promote mitochondrial biogenesis in microglia.** (**A**) Representative western blot image of PGC1-α in BV2 microglia treated with or without 50 µM QA for 72 h. β-actin was used for loading control. (**B**) Quantification of relative protein level of PGC1-α in **A** (*n* = 3). Data are means ± SEM.


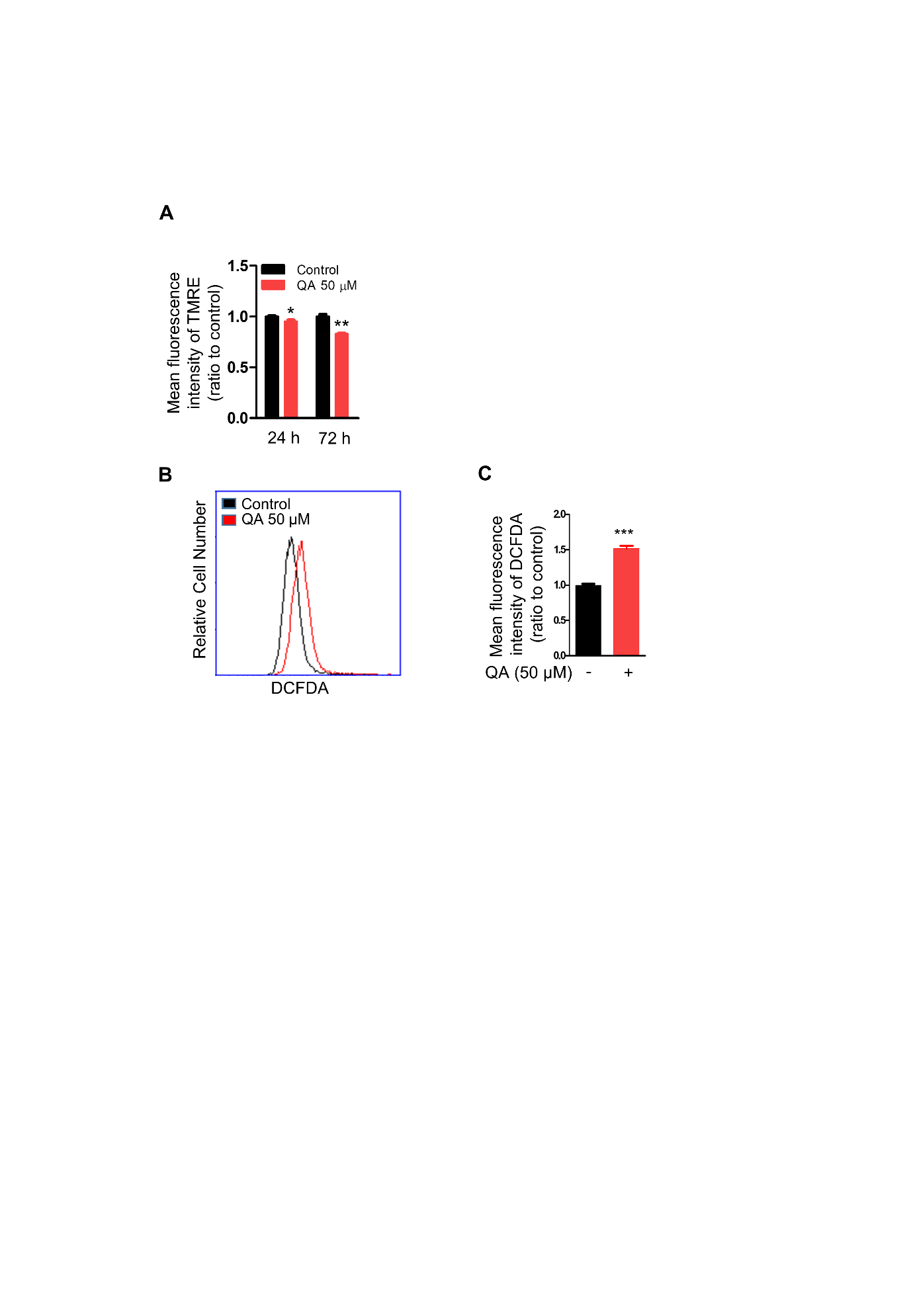


**Fig. S3. QA induces mitochondrial dysfunction in microglia.** (**A**) Flow cytometric analysis for MMP in BV2 microglia treated with or without 50 µM QA for the indicated time using TMRE dye. Graph represents the mean fluorescence intensity of TMRE (*n* = 3-5). (**B**) Flow cytometric analysis for cellular ROS generation in BV2 microglia treated with or without
50 µM QA for 72 h using DCFDA. Histograms were used to determine cellular ROS in BV2 microglia. (**C**) Graph represents the mean fluorescence intensity of DCFDA (*n* = 5). Data are means ± SEM. * *p* < 0.05, ** *p* < 0.01 and *** *p* < 0.001 versus no treatment (control) group; Two-tailed unpaired *t* test (**A** and **C**).


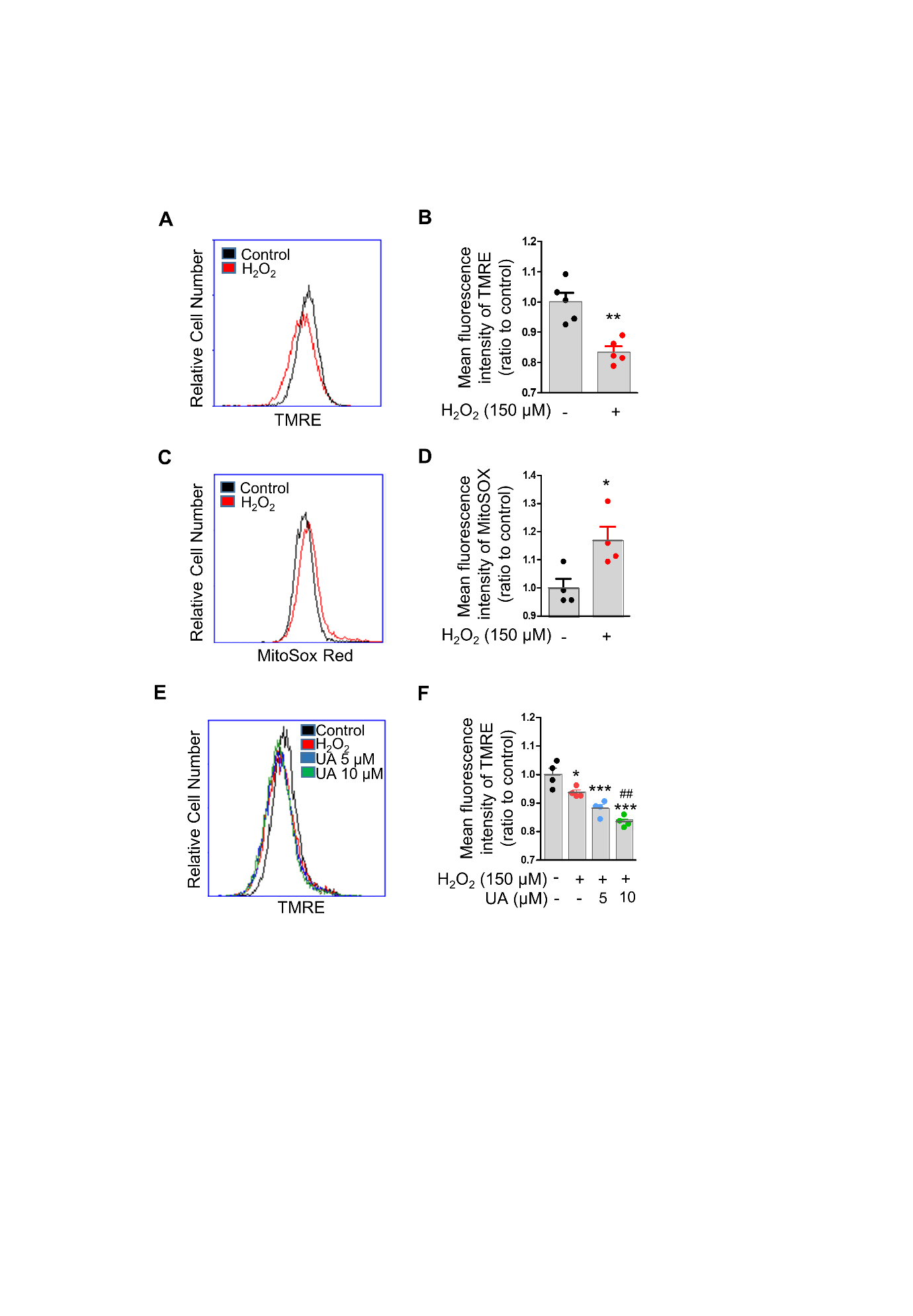


**Fig. S4. Oxidative stress induces mitochondrial dysfunction in microglia.** (**A**) Flow cytometric analysis for MMP in BV2 microglia treated with or without 150 µM H_2_O_2_ for 72 h using TMRE dye. Histograms were used to determine MMP in BV2 microglia. (**B**) Graph represents the mean fluorescence intensity of TMRE (*n* = 5). (**C**) Flow cytometric analysis for mitochondrial ROS generation in BV2 microglia treated with or without 150 µM H_2_O_2_ for 72 h using MitoSOX. Histograms were used to determine mitochondrial ROS in BV2 microglia. (**D**) Graph represents the mean fluorescence intensity of MitoSOX (*n* = 4). (**E**) Flow cytometric analysis for MMP in BV2 microglia treated with or without 150 µM H_2_O_2_ in the absence or presence of UA as indicated for 72 h using TMRE dye. Histograms were used to determine MMP in BV2 microglia. (**F**) Graph represents the mean fluorescence intensity of TMRE (*n* = 4). Data are means ± SEM. * *p* < 0.05, ** *p* < 0.01 and *** *p* < 0.001 versus no treatment (control) group; ^##^ *p* < 0.01 versus H_2_O_2_ alone treatment group; Two-tailed unpaired *t* test (**B** and **D**) or one-way ANOVA followed by Tukey’s multiple comparisons test (**F**).


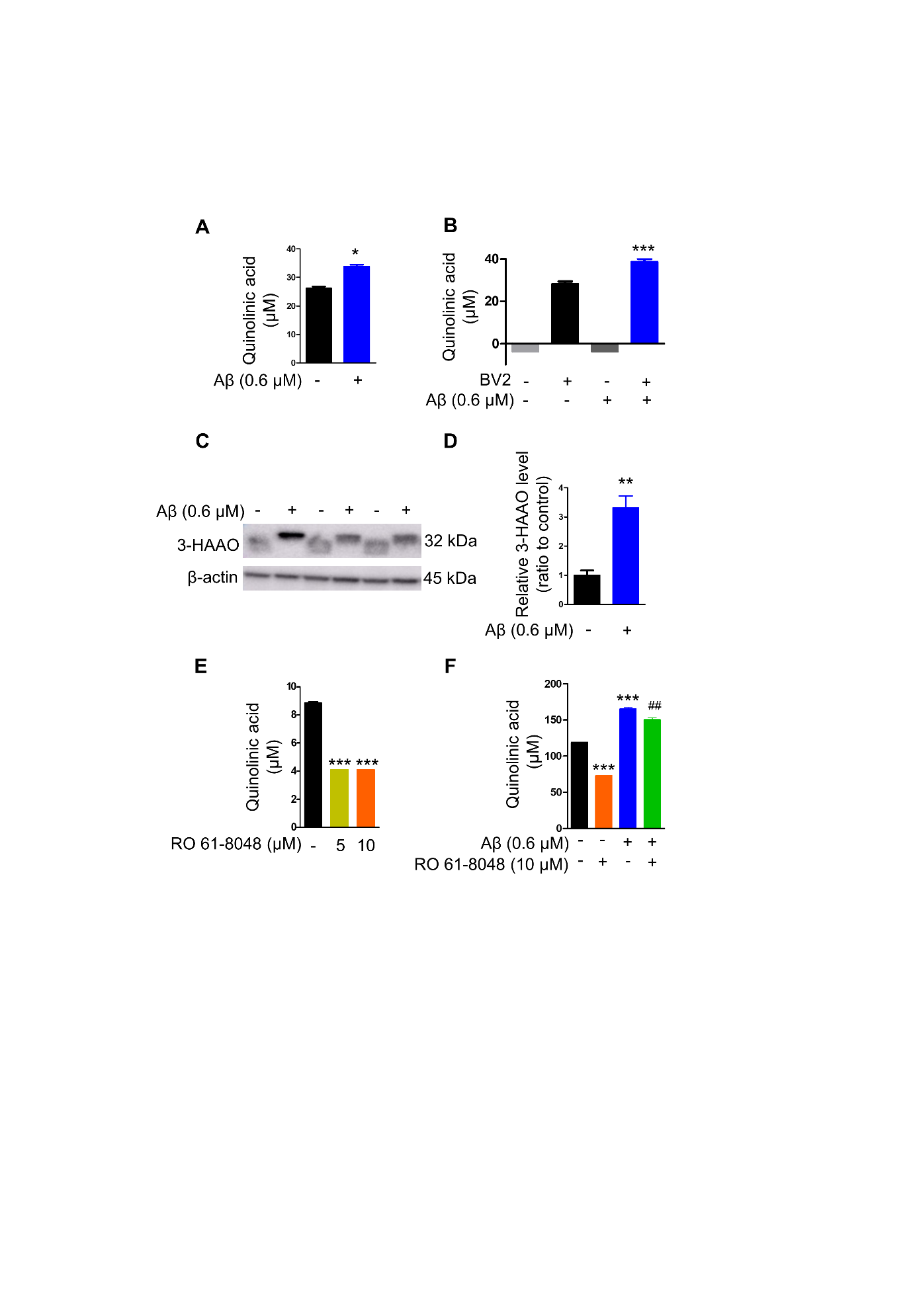


**Fig. S5. QA level increases after Aβ_1-42_ stimulation in microglia.** (**A**) HPLC analysis of QA in the conditioned medium obtained from BV2 microglia treated with or without 0.6 µM
Aβ_1-42_ for 48 h (*n* = 3). (**B**) HPLC analysis of QA in the conditioned medium obtained from BV2 microglia treated with or without 0.6 µM Aβ_1-42_ for 48 h in the absence or presence of primary hippocampal neurons (DIV14) (*n* = 4-6). (**C**) Representative western blot image of
3-HAAO in primary hippocampal neurons (DIV14) and BV2 microglia co-culture treated with or without 0.6 µM Aβ_1-42_ for 48 h. β-actin was used for loading control. (**D**) Quantification of relative protein level of 3-HAAO in **C** (*n* = 3). (**E**) HPLC analysis of QA in the conditioned medium obtained from BV2 microglia treated with or without RO 61-8048 as indicated for 48 h (*n* = 3). (**F**) HPLC analysis of QA in the conditioned medium obtained from BV2 microglia and primary hippocampal neurons (DIV14) co-culture treated with or without 0.6 µM Aβ_1-42_ for 48 h in the absence or presence of 10 µM RO 61-8048 (*n* = 3). HPLC analysis was carried out using mobile phase of 25% methanol : 75% 10 mM sodium dihydrogen phosphate at final pH 2.0, a flow rate of 1.15 ml/min at 37 °C with UV (220 nm). Injection volume was 10 µl. Data are means ± SEM; * *p* < 0.05, ** *p* < 0.01 and *** *p* < 0.001 versus no treatment (control) group, ^##^ *p* < 0.01 versus Aβ_1-42_ alone treatment group; Two-tailed unpaired *t* test (**A** and **D**) or one-way ANOVA followed by Tukey’s multiple comparisons test (**B, E** and **F**).
