# Supplementary material for: Quinolinic acid impairs mitophagy promoting microglia senescence and poor healthspan in C. elegans: a mechanism of impaired aging process
Source: Biol Direct. 2023 Dec 20;18:86. doi: 10.1186/s13062-023-00445-y (PMC10734169; doi:10.1186/s13062-023-00445-y)
Supplement: Supplementary file 2 — Additional File 2: Western blots full scans [file 13062_2023_445_MOESM2_ESM.pptx]

## Slide 1
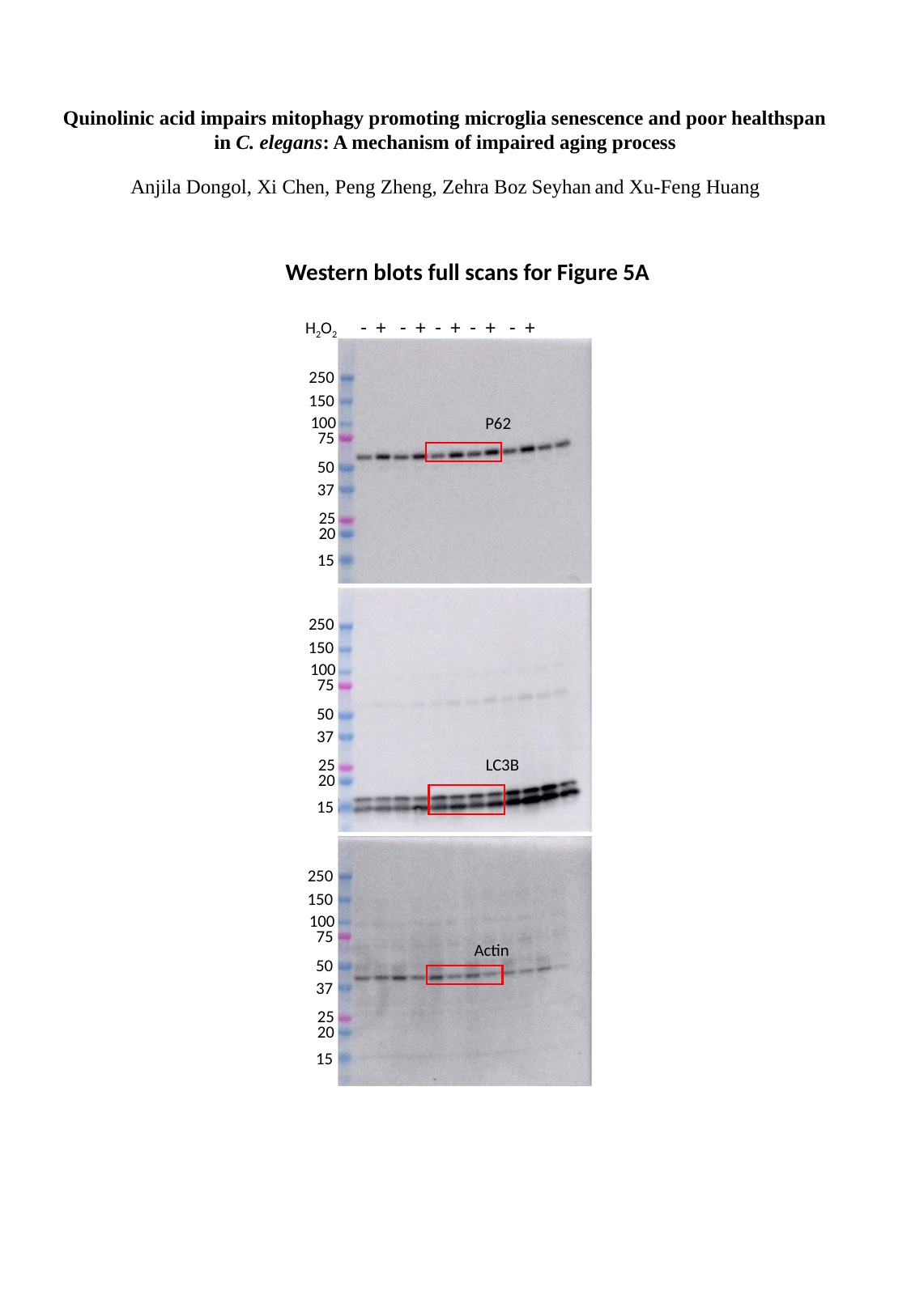

Quinolinic acid impairs mitophagy promoting microglia senescence and poor healthspan in C. elegans: A mechanism of impaired aging process
Anjila Dongol, Xi Chen, Peng Zheng, Zehra Boz Seyhan and Xu-Feng Huang
Western blots full scans for Figure 5A
- + - + - + - + - +
H2O2
250
150
100
P62
75
50
37
25
20
15
250
150
100
75
50
37
LC3B
25
20
15
250
150
100
75
Actin
50
37
25
20
15

## Slide 2
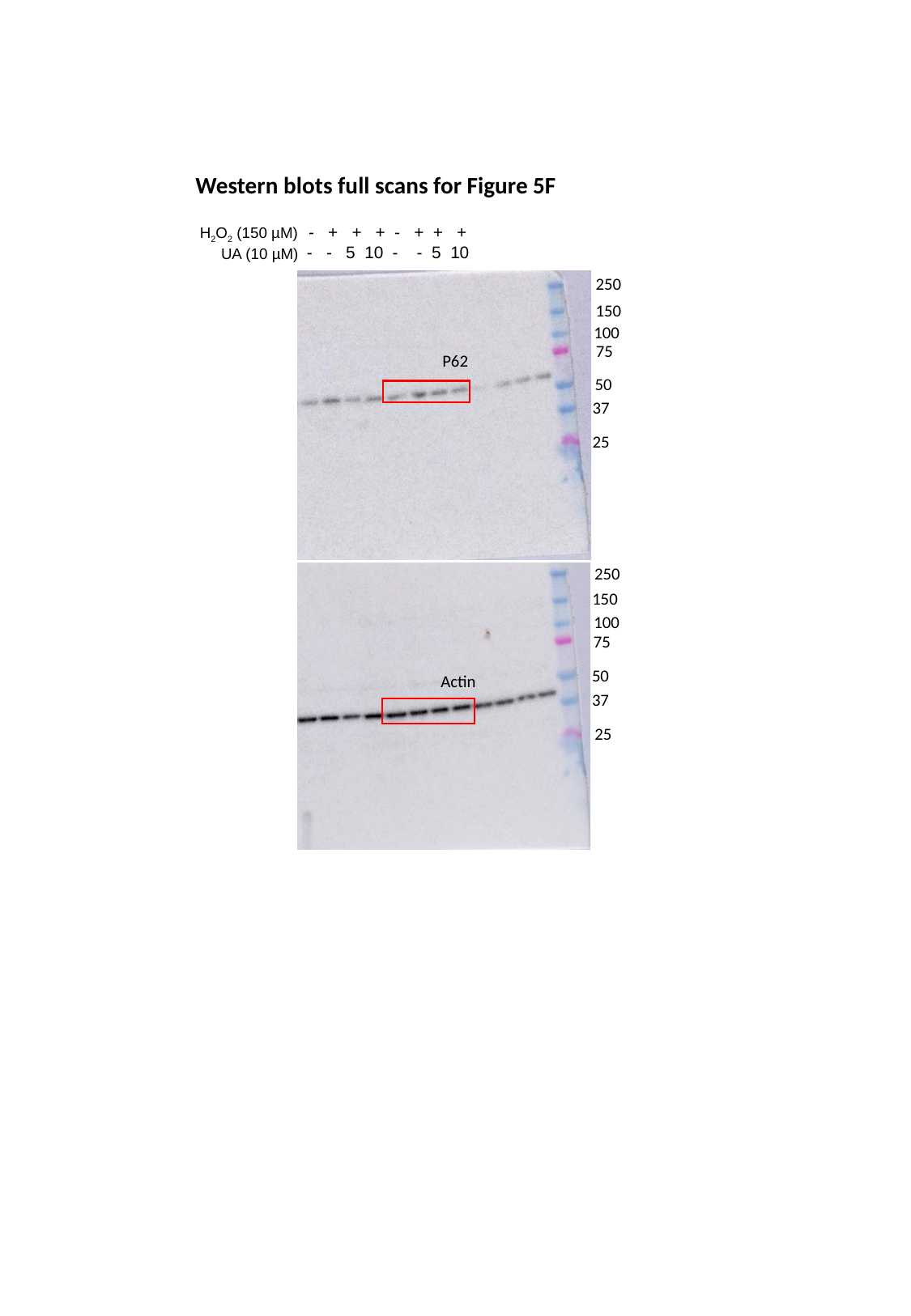

Western blots full scans for Figure 5F
- + + + - + + +
H2O2 (150 µM)
- - 5 10 - - 5 10
UA (10 µM)
250
150
100
75
P62
50
37
25
250
150
100
75
50
Actin
37
25

## Slide 3
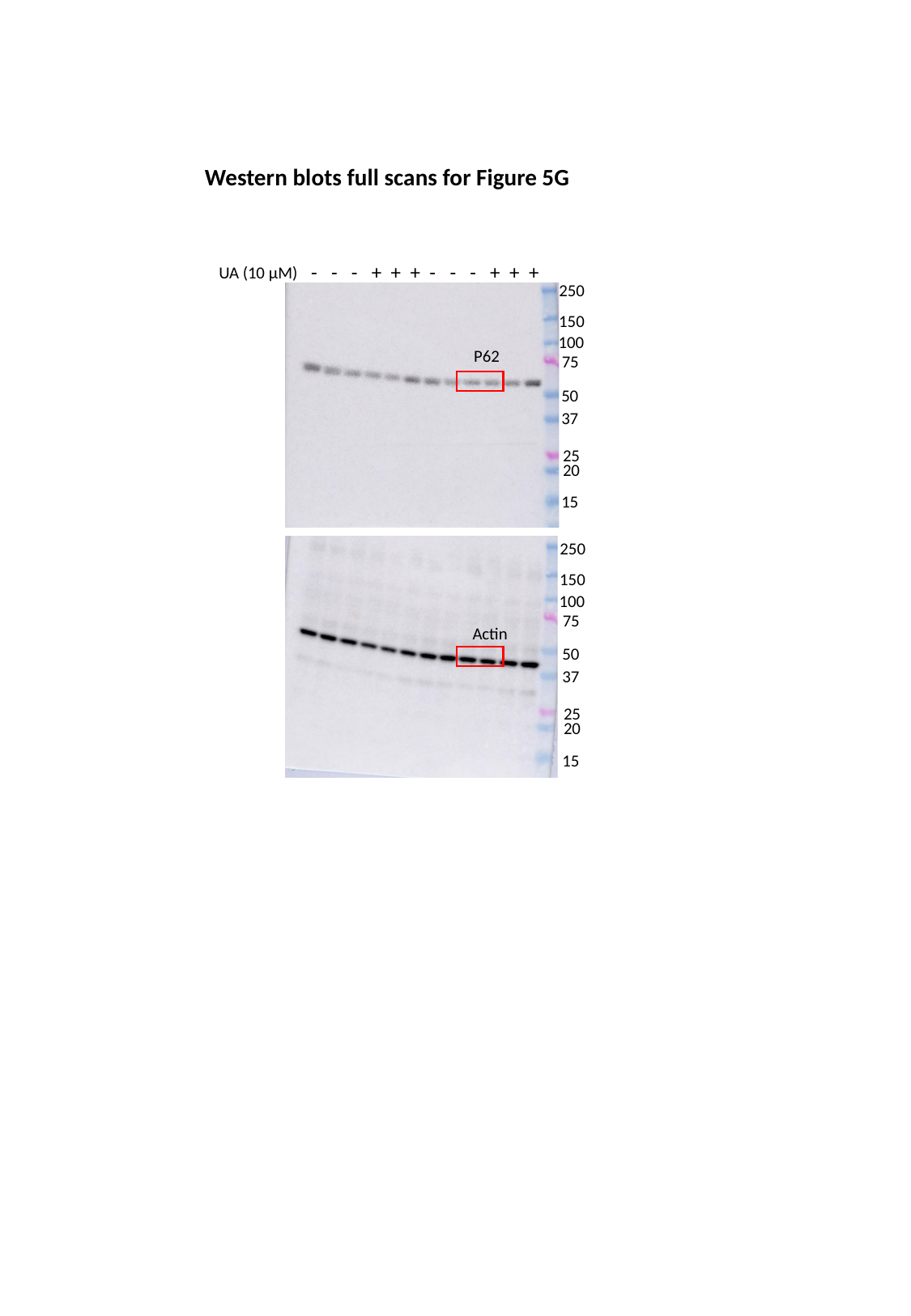

Western blots full scans for Figure 5G
- - - + + + - - - + + +
UA (10 µM)
250
150
100
P62
75
50
37
25
20
15
250
150
100
75
Actin
50
37
25
20
15

## Slide 4
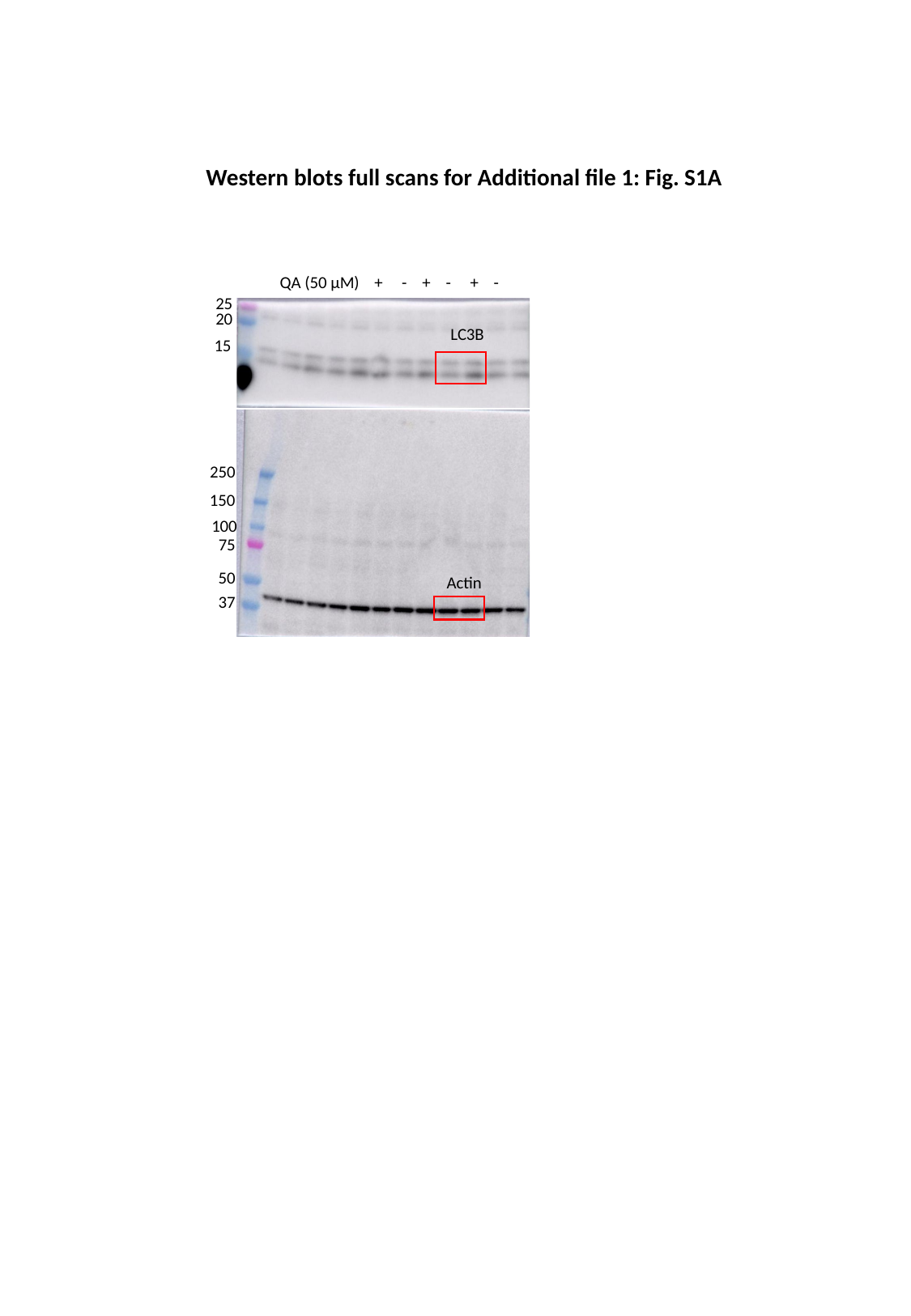

Western blots full scans for Additional file 1: Fig. S1A
QA (50 µM)
 + - + - + -
25
20
LC3B
15
250
150
100
75
50
Actin
37

## Slide 5
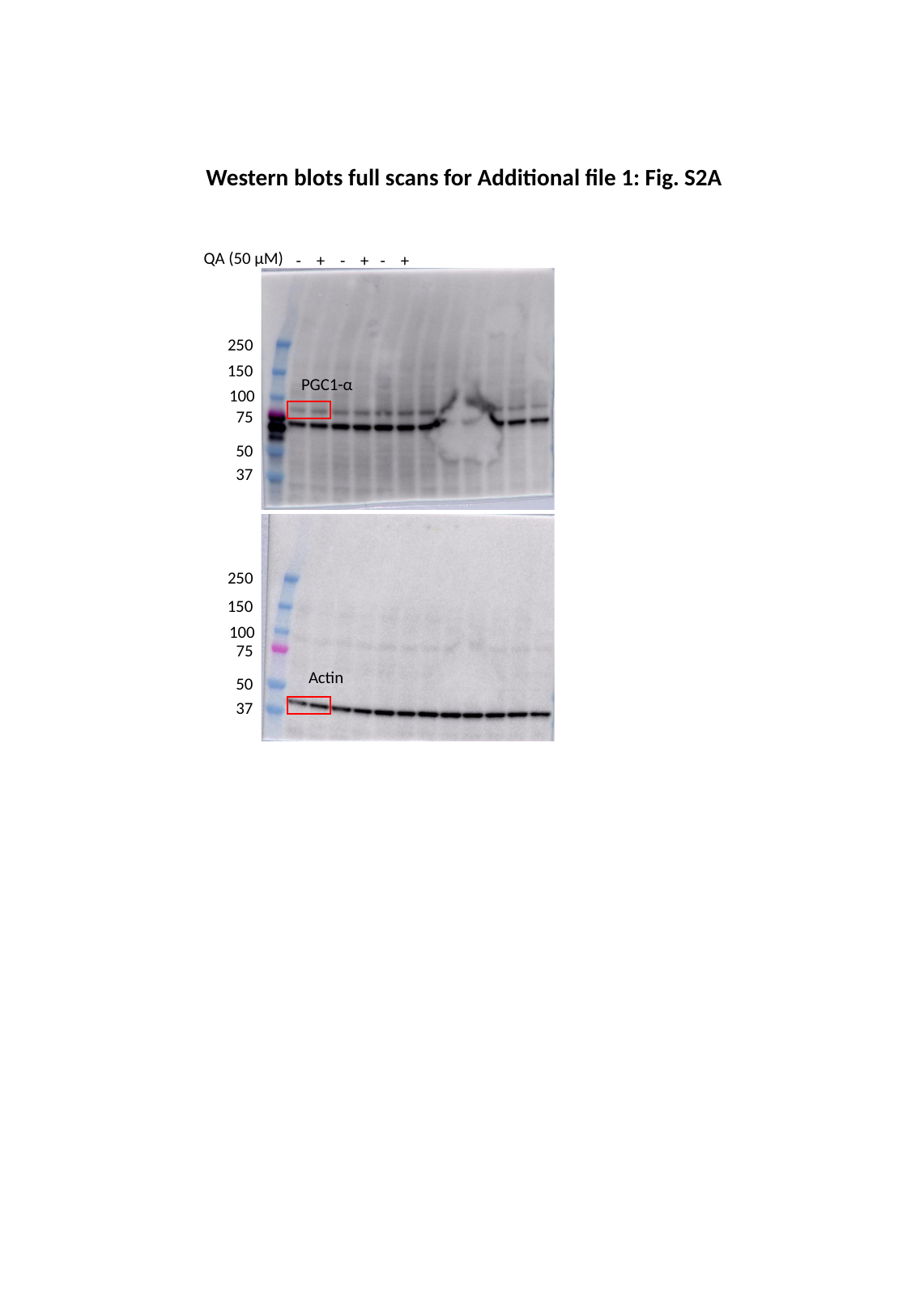

Western blots full scans for Additional file 1: Fig. S2A
QA (50 µM)
 - + - + - +
250
150
PGC1-α
100
75
50
37
250
150
100
75
Actin
50
37

## Slide 6
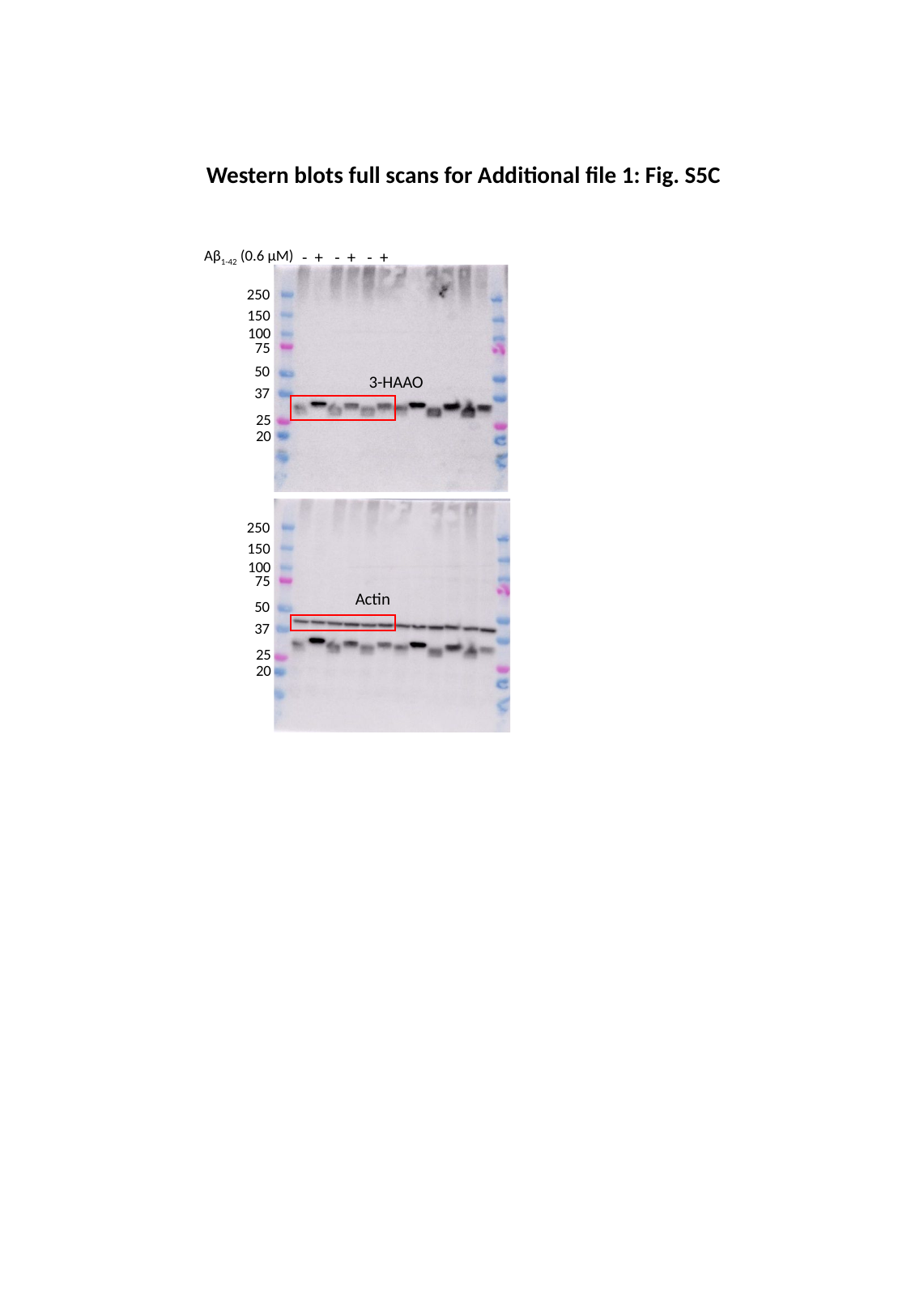

Western blots full scans for Additional file 1: Fig. S5C
Aβ1-42 (0.6 µM)
 - + - + - +
250
150
100
75
50
3-HAAO
37
25
20
250
150
100
75
Actin
50
37
25
20
